# Oral Celastrol Micelles Forming High‐Density Lipoprotein Corona Targeting Hepatocytes for MASLD Treatment

**DOI:** 10.1002/advs.202500854

**Published:** 2025-09-23

**Authors:** Chang Xu, Haoru Zhu, Kai Wang, Haitao Hu, Xuyong Wei, Dongdong Xu, Jing Zhang, Yanpeng Liu, Jun Chen, Youqing Shen, Nasha Qiu, Xiao Xu

**Affiliations:** ^1^ Hangzhou First People’s Hospital Zhejiang University School of Medicine Hangzhou 310058 China; ^2^ Key Laboratory of Integrated Oncology and Intelligent Medicine of Zhejiang Province Affiliated Hangzhou First People's Hospital School of Medicine Westlake University Hangzhou 310006 China; ^3^ Zhejiang University School of Medicine Hangzhou 310058 China; ^4^ Key Laboratory of Smart Biomaterials of Zhejiang Province and Key Laboratory of Biomass Chemical Engineering of the Ministry of Education of China College of Chemical and Biological Engineering Zhejiang University Hangzhou 310058 China; ^5^ School of Clinical Medicine Hangzhou Medical College Hangzhou 310053 China; ^6^ Hepatobiliary Center The First Affiliated Hospital of Nanjing Medical University Nanjing 210000 China

**Keywords:** celastrol, HDL corona, liver‐targeting micelles, MASLD

## Abstract

Metabolic dysfunction‐associated steatotic liver disease (MASLD) is a multifactorial hepatic manifestation of metabolic syndrome, such as aberrant lipid accumulation. Celastrol (CEL), a potent leptin sensitizer, has been studied for the treatment of MASLD; however, its clinical application is hindered by its low oral bioavailabilities and high toxicities. Herein, CEL is encapsulated into the micelles of poly[2‐(N‐oxide‐N,N‐diethylamino)ethyl methacrylate]‐block‐poly(ε‐caprolactone) (OPDEA‐PCL), whose shell is capable of fast penetrating mucus due to its protein‐non‐fouling and rapid transcytosis induction as a result of phospholipid‐binding characteristic. After oral administration, OPDEA‐PCL/CEL micelles sequentially permeated through the gastrointestinal barriers into the blood. Notably, it is found that OPDEA‐PCL/CEL specifically captured high‐density lipoproteins (HDL) in plasma, forming an HDL corona actively targeting the hepatocytes, delivering a high concentration of CEL. In the MASLD mouse model, oral administration of OPDEA‐PCL/CEL effectively alleviated hepatic lipid accumulation, lessened hepatic inflammation, and mitigated the toxicities of CEL with therapeutic efficacy superior to free CEL and PEG‐PCL/CEL and even the current clinical simvastatin. Therefore, the OPDEA‐PCL may be a promising oral CEL delivery system for treating MASLD.

## Graphical abstract

Upon oral administration, OPDEA‐PCL/CEL micelles passed through the stomach acid intact, then quickly penetrated the intestinal mucus and transported into circulation, where they captured HDL to form a protein corona and target hepatocyte SR‐B1 receptors, inducing endocytosis. Then CEL released from micelles, modulating lipid metabolism and reducing lipid accumulation of steatotic liver.

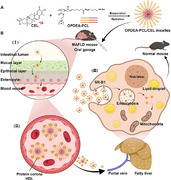

## 1 Introduction

Metabolic dysfunction‐associated steatotic liver disease (MASLD), recognized as the hepatic manifestation of metabolic syndrome, has reached epidemic proportions due to its close link with obesity.^[^ ^1^ ^]^ It is estimated that the global incidence of MASLD is ≈ 30% among adults, posing a significant global health challenge.^[^ ^2^ ^]^ It is characteristic of abnormal buildup of lipids within the liver and the imbalance of lipid metabolism, which results in the production of lipotoxic lipids that induce cellular stress and subsequent stimulation of inflammation.^[^ ^3^ ^]^ In liver transplantation, donor liver shortage remains a critical global issue.^[^ ^4^ ^]^ With the ongoing increase in obesity prevalence globally, an increased incidence of hepatic steatosis among organ donors is expected.^[^ ^5^ ^]^ It is reported that donor livers with hepatic steatosis account for 39% of organ discard,^[^ ^6^ ^]^ as utilizing these fatty livers can elevate the risk of graft failure by 53%.^[^ ^7^ ^]^ Recently, the liver donor shortage has led to an increasing utilization of steatosis liver grafts, elevating the risk of primary non‐function, early allograft dysfunction, and diminished long‐term patient outcomes.^[^ ^6^, ^8^ ^]^

MASLD is generally treated with physical activity and a healthy diet. Recently, a PPAR pan agonist, lanifibranor, is approved in phase III trial for treating MASLD.^[^ ^9^ ^]^ However, due to serious adverse events, the clinical trial protocol was adjusted, and the recruitment of new patients was temporarily halted. Thus, the development of oral medications for treating steatotic liver is in urgent demand.

Celastrol (CEL), a natural product originating from traditional Chinese medicine, has been tested as a potential therapeutic agent for metabolic diseases, especially obesity and diabetes.^[^ ^10^ ^]^ Particularly, CEL was identified as a leptin sensitizer;^[^ ^11^ ^]^ the treated mice had reduced liver weights due to decreased hepatic steatosis and intrahepatic triglyceride accumulation.^[^ ^12^ ^]^ However, CEL, characterized by its high hydrophobicity and poor aqueous solubility, results in limited intestinal absorption and bioavailability.^[^ ^13^ ^]^ Meanwhile, it may cause organ aberrance, including reproductive disorders, cardiotoxicity, hepatotoxicity, hematopoietic system abnormality.^[^ ^14^ ^]^ CEL was encapsulated into poly(ethylene glycol)‐poly(ε‐caprolactone) copolymers (PEG‐PCL)^[^ ^15^ ^]^ or conjugated to bovine serum albumin (BSA)^[^ ^16^ ^]^ to reduce toxicities and improve biocompatibility. However, these delivery systems failed to overcome the gastrointestinal delivery barriers, suffering low oral bioavailabilty and inefficient hepatic targeting.^[^ ^17^ ^]^

**Scheme 1:**
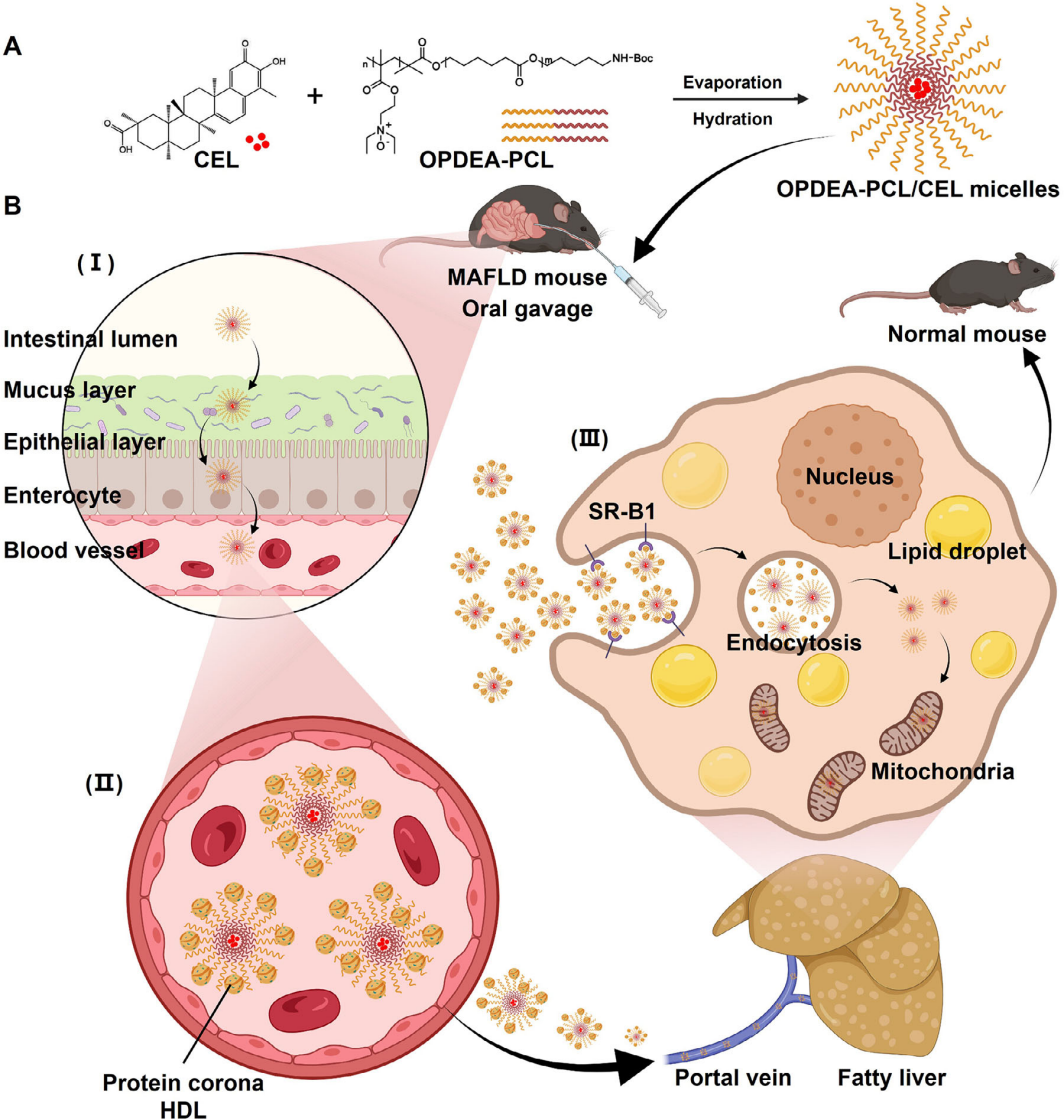
The schematic illustration demonstrates the treatment of MASLD through oral administration of OPDEA‐PCL/CEL micelles (Created in BioRender. https://BioRender.com/vcv71p3). A) Structures of the CEL and OPDEA‐PCL polymer, and their self‐assembly for CEL encapsulation into the OPDEA‐PCL micelles. B) The treatment of MASLD with the micelles. Upon oral administration, the OPDEA‐PCL/CEL micelles effectively navigated through the acidic gastric environment, maintaining stability during the transportation to the intestine, where it rapidly permeated through the intestinal mucus layer (I). The micelles were transported by intestinal epithelial cells via transcytosis into the intestinal venous circulation, where they captured HDLs onto their surface to form an HDL protein corona (II) that actively targeted the hepatocyte SR‐B1 receptors and triggered endocytosis (III). CEL was then released to modulate lipid metabolism and reduce lipid accumulation, subsequently improving the hepatocyte function.

Oral administration is clinic‐favored due to its high patient compliance in repeated dosing.^[^ ^18^ ^]^ However, the necessity to surmount a range of anatomical and physiological hurdles within the gastrointestinal tract (GIT) remains; thus only few delivery systems can address the sophisticated physiological conditions.^[^ ^19^ ^]^ We found that OPDEA‐PCL effectively penetrated mucus and rapidly crossed the intestinal epithelial barrier, achieving high oral availability.^[^ ^20^ ^]^ Here, we further discovered that OPDEA‐PCL micelles actively adsorbed high‐density lipoprotein (HDL) in plasma, forming an HDL corona that enabled active liver‐targeting.^[^ ^21^ ^]^ The underlying mechanism was that the OPDEA shell was non‐fouling to proteins such as albumin^[^ ^22^ ^]^ but selectively bound the phospholipids in the HDL; thus the OPDEA‐PCL micelles adsorbed HDL via its phospholipids, exposing the HDL's protein Apo‐A1,^[^ ^23^ ^]^ which can be recognized by the hepatocytes’ scavenger receptor SR‐B1 triggering endocytosis.^[^ ^24^ ^]^ Thus, the orally administered OPDEA‐PCL/CEL rapidly entered the systemic circulation and then the liver via the portal vein and actively targeted hepatocytes. In a MASLD mouse model, OPDEA‐PCL/CEL significantly reduced the average body weight by over 20%, decreased pathological lipid droplet accumulation in hepatocytes by more than 90%, attenuated hepatic inflammation, and mitigated the toxicities of CEL. The therapeutic efficacy of OPDEA‐PCL/CEL micelles was superior to free CEL and PEG‐PCL/CEL and current clinical drug simvastatin, suggesting that OPDEA‐PCL/CEL system could be a viable therapeutic candidate for the treatment of MASLD. **Scheme** **1**.

## 2 Results and Discussion

### 2.1 Synthesis, Characterization, and Cytotoxicity of OPDEA‐PCL/CEL Micelles

**Figure 1:**
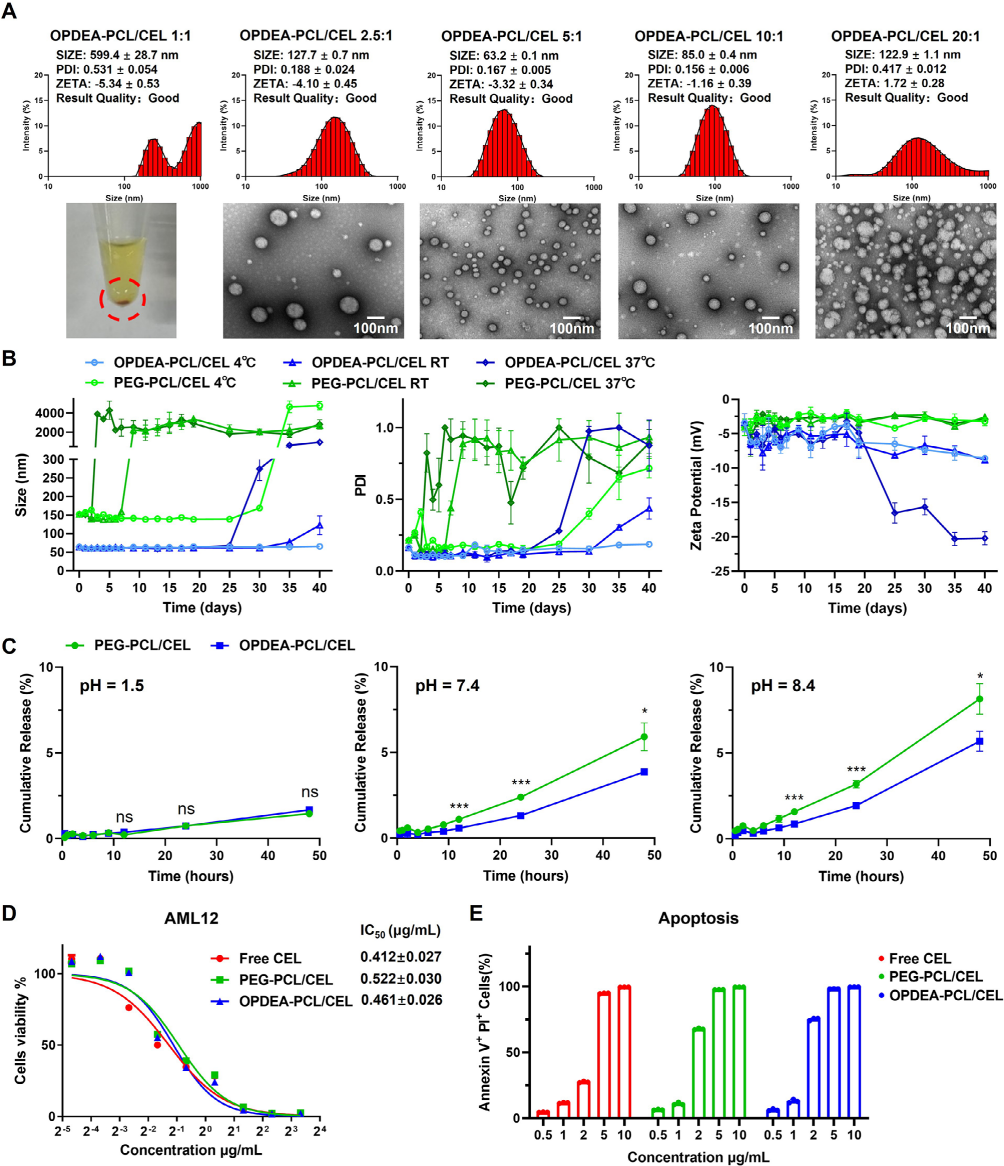
Characterization and cytotoxicities of OPDEA‐PCL/CEL and PEG‐PCL/CEL micelles on AML12 cells. A) The sizes and morphologies of OPDEA‐PCL/CEL micelles at different mass ratios were characterized by dynamic light scattering (DLS) and transmission electron microscopy (TEM). At 1:1 mass ratio, stable micelles did not form but precipitated (circled in red). B) The stabilities of OPDEA‐PCL/CEL and PEG‐PCL/CEL micelles were assessed by monitoring the changes in particle sizes, size distributions (PDIs) and zeta potentials following timed storage at 4 °C, room temperature (RT) or 37 °C. C) The release profiles of CEL from the micelles (CEL‐eq. 0.2 mg mL^−1^) in the simulated physiological media (simulated intestinal fluid pH 8.4 and simulated gastric fluid pH 1.5) and PBS (pH 7.4). D) Cytotoxicity of free CEL, OPDEA‐PCL/CEL and PEG‐PCL/CEL micelles on AML12 cells after incubation for 24 h. (E) Flow cytometry assessment of apoptosis (Annexin V^+^ PI^+^ cells) in AML12 cells after incubation with free CEL, OPDEA‐PCL/CEL and PEG‐PCL/CEL micelles for 24 h. Data are expressed as the mean ± standard deviation from three biological replicates.

The OPDEA‐PCL was synthesized and characterized according to previous studies,^[^ ^20a^ ^]^ as shown in Figures S1–S6 (Supporting Information). The PCL segment was controlled to be ≈ 5 kDa, with the OPDEA segment varied to achieve block ratios of OPDEA to PCL at 2:5, 3:5, 4:5, 5:5, 6:5, and 8:5, respectively. CEL was self‐assembled with OPDEA‐PCL using a thin‐film hydration technique. The control group, PEG‐PCL/CEL micelles, was prepared by the same method. The block ratio of OPDEA‐PCL was optimized to be 5KDa:5 KDa according to the size distribution (Figure S7, Supporting Information). The mass ratio of OPDEA‐PCL to CEL was varied from 1:1 to 20:1. At the 5:1 ratio, the formed OPDEA‐PCL/CEL micelles had the most uniform particle size so they were selected for subsequent experiments. The averaged diameter of the OPDEA‐PCL/CEL micelles was 63.2 ± 0.1 nm. TEM image showed that OPDEA‐PCL/CEL micelles had a spherical morphology (**Figure** **1**A). The critical micelle concentrations (CMC) of OPDEA‐PCL and PEG‐PCL were 19.04 ± 0.18  and 33.30 ± 0.29 µg mL^−1^, respectively (Figure S8, Supporting Information). The drug loading content and encapsulation efficiency were 8.10% and 89.00%, as analyzed by HPLC.

Long‐term stability assessments revealed that OPDEA‐PCL/CEL micelles exhibited negligible alterations in particle size and size distribution within 4 weeks and were relatively stable at 4 °C and room temperature, but the particles disassembled within 3 days at 37 °C (Figure 1B; Figure S9, Supporting Information). We also tested the integrity and stability of these micelles in PBS (pH 7.4) and the simulated physiological media, including simulated intestinal fluid (SIF, pH 8.4) and simulated gastric fluid (SGF, pH 1.5). OPDEA‐PCL/CEL micelles and PEG‐PCL/CEL micelles exhibited excellent stabilities at pH 7.4 and 1.5, with no significant changes in CEL release and particle sizes. At pH 8.4, OPDEA‐PCL/CEL micelles demonstrated a slow release of CEL, with a cumulative release of 5% of the encapsulated CEL release within 48 h, more stable than PEG‐PCL/CEL micelles (released ≈ 10%) (Figure 1C; Figure S10, Supporting Information).

The cytotoxicity of free CEL, PEG‐PCL/CEL micelles and OPDEA‐PCL/CEL micelles were evaluated on AML12 and HepG2 cells using the CCK‐8 assay. The calculated IC_50_ values were 0.41 ± 0.03 µg/mL for free CEL, 0.52 ± 0.03 µg mL^−1^ for PEG‐PCL/CEL micelles, and 0.46 ± 0.03 µg mL^−1^ for OPDEA‐PCL/CEL micelles in AML12 cells, respectively (Figure 1D). Similar results were noted in HepG2 cells (Figure S11, Supporting Information). These findings indicated that the cytotoxicity of these three were comparable, also confirmed by apoptosis analysis (Figure 1E; Figure S12, Supporting Information).

### 2.2 OPDEA‐PCL/CEL Micelles Enhanced Cellular Uptake and Reduced Lipid Accumulation in Hepatocytes Through Mitochondria Colocalization

**Figure 2:**
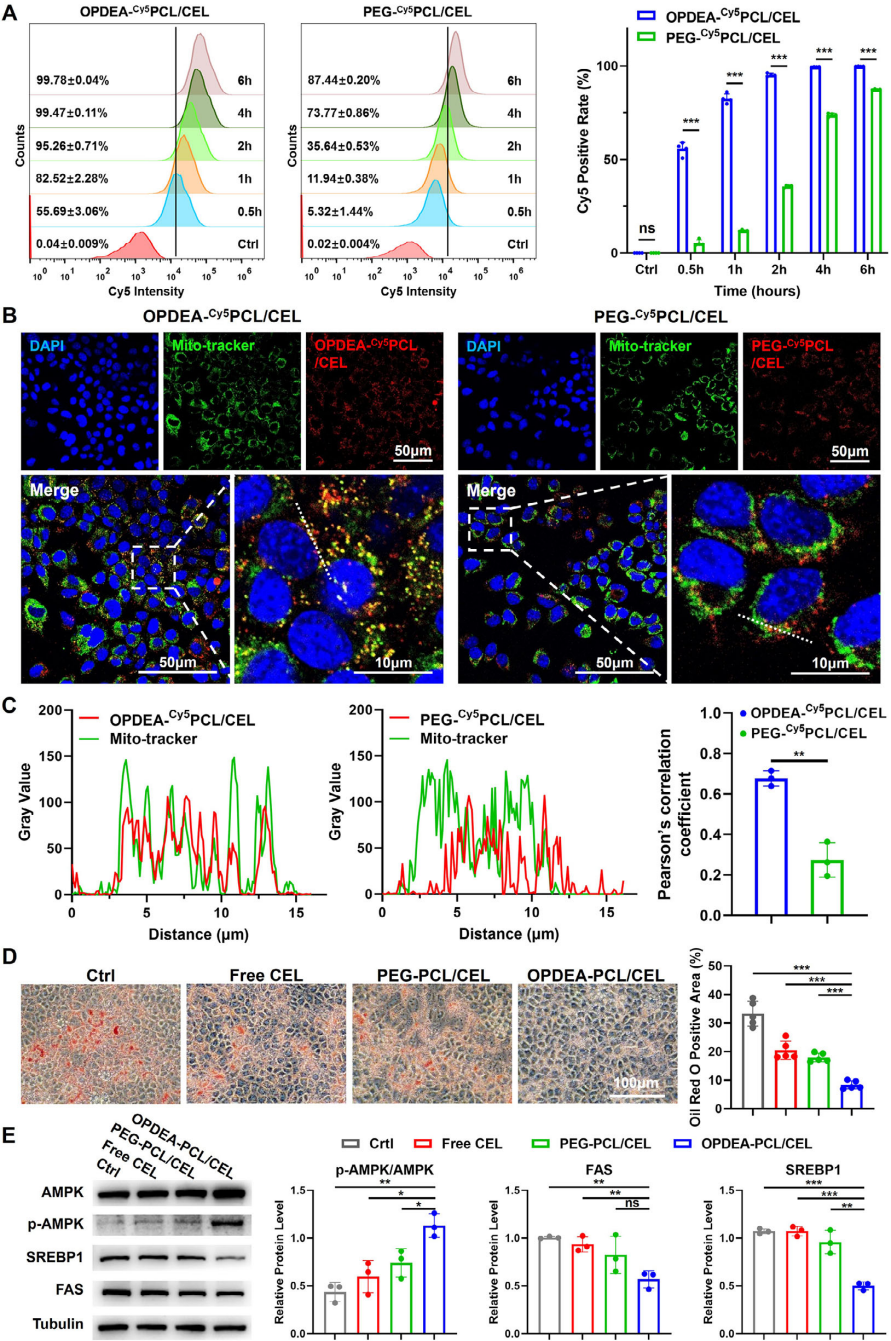
Hepatocyte uptake and steatosis reduction of OPDEA‐PCL/CEL and PEG‐PCL/CEL micelles tested on AML12 cells. A) The flow cytometry profiles and the corresponding quantitation of Cy5‐positive cells of AML12 cells treated with OPDEA‐^Cy5^PCL/CEL or PEG‐^Cy5^PCL/CEL micelles (Cy5‐equivalent dose of 0.5 µg mL^−1^) for timed intervals (*n* = 4). B) Mitochondrial colocalization analysis of AML12 cells incubated with Cy5‐labeled micelles (Cy5‐ equivalent dose of 0.5 µg mL^−1^) for 6 h measured by Laser Scanning Confocal Microscope (LSCM). C) Line scan analysis and Pearson's correlation coefficient calculation along three randomly selected lines in the LSCM images in B using ImageJ software (version 1.52i) (*n* = 3). D) Representative images and quantitation of lipid accumulation by Oil Red O staining in AML12 cells after incubation with oleate (OA) and treated with free CEL, OPDEA‐PCL/CEL, or PEG‐PCL/CEL micelles (CEL‐eq. 0.5 µg mL^−1^) for 48 h. Lipid droplet area was assessed by five randomly selected fields through ImageJ software (version 1.52i). E) Western blot analysis and quantitation of AMPK, p‐AMPK, SREBP1 and FAS expression of AML12 cells after treated with free CEL, OPDEA‐PCL/CEL, or PEG‐PCL/CEL micelles (CEL‐eq. 0.5 µg mL^−1^) for 48 h (*n* = 3). Data are expressed as the mean ± standard deviation from biological replicates.

The unique interaction between OPDEA polymers and phospholipids^[^ ^22^ ^]^ facilitates the fast adsorption‐mediated endocytosis of OPDEA‐PCL micelles in comparison to PEG‐PCL micelles, which were internalized by cells slowly.^[^ ^25^ ^]^ We investigated the cellular uptake behaviors of these two micelles through endocytosis assays. The micelles were labeled with cyanine 5 (Cy5). Flow cytometry analysis showed that OPDEA‐^Cy5^PCL/CEL micelles were internalized by AML12 cells at a significantly faster rate compared to PEG‐based micelles. Within 1‐h, 80% of the cells incubated with OPDEA‐^Cy5^PCL/CEL already took up measurable Cy5, compared to 11.9% of those cultured with PEG‐^Cy5^PCL/CEL micelles. PEG‐^Cy5^PCL/CEL micelles required 6 h to enter most cells (**Figure** **2**A; Figure S13, Supporting Information).

Previous studies demonstrated that CEL mediated lipid metabolism through mitochondria.^[^ ^26^ ^]^ OPDEA is mitochondria‐philic,^[^ ^27^ ^]^ so we hypothesized that OPDEA might directly deliver CEL to mitochondria and thus enhance CEL's lipid metabolism regulation. As seen from Figure 2B, after 6‐h incubation with AML12 cells, OPDEA‐^Cy5^PCL/CEL colocalized extensively with mitochondria, whereas the majority of PEG‐^Cy5^PCL/CEL was distributed within cells. The Pearson correlation factors of OPDEA‐^Cy5^PCL/CEL with mitochondria was 0.68, ≈ 3‐fold that of PEG‐^Cy5^PCL/CEL (0.26) (Figure 2C).

The effects of OPDEA‐PCL/CEL on cellular lipid metabolism were assessed in hepatic steatosis AML12 cells. The Oil Red O staining showed that oleate (OA) treatment caused intracellular lipid accumulation, while the lipid contents were decreased in cells treated with free CEL, PEG‐PCL/CEL, or OPDEA‐PCL/CEL. Notably, OPDEA‐PCL/CEL treatment was the most efficient, decreasing from 33.3% to 8.3%. In contrast, CEL and PEG‐PCL/CEL only reduced to 17.9% (Figure 2D). Western‐blot analyses of lipid metabolic pathways revealed that OPDEA‐PCL/CEL increased the phosphorylated AMPK to total AMPK ratios, downregulated FAS and SREBP1 expression (Figure 2E), while CEL and PEG‐PCL/CEL were much less effective. Thus, OPDEA‐PCL/CEL micelles’ rapid cellular internalization and mitochondria‐targeted delivery may account for the reduction of cellular steatosis.

### 2.3 OPDEA‐PCL/CEL Micelles Effectively Penetrated Mucus and Crossed Epithelium

**Figure 3:**
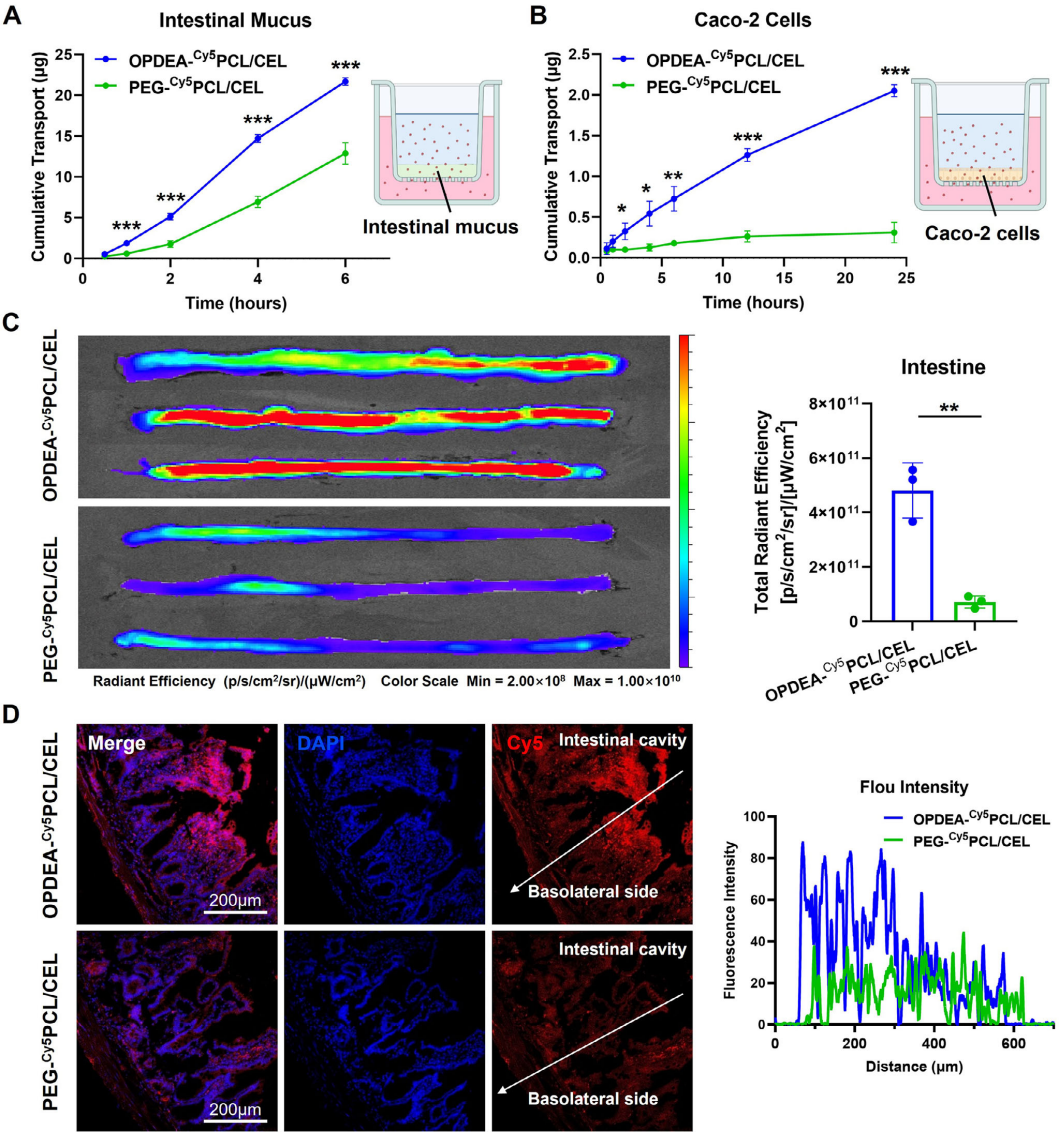
Mucus penetration and intestinal absorption of OPDEA‐PCL/CEL and PEG‐PCL/CEL micelles. A) Measurement of the transmucosal transport of Cy5‐labeled micelles through a 500‐µm‐thick mucus layer of rat intestine coated on a Transwell insert (micropore size ≈ 0.4 µm). 200 microliters of OPDEA‐^Cy5^PCL/CEL or PEG‐^Cy5^PCL/CEL solution (Cy5‐eq. dose, 10 µg mL^−1^) were added to the apical compartment. The Cy5 fluorescence intensity was measured at timed intervals to determine the concentrations of the micelles in the basolateral compartment. B) Measurement of the capability of Cy5‐labeled micelles to undergo transepithelial movement through a monolayer of Caco‐2 cell coated on a Transwell insert (micropore size ≈ 0.4 µm). The Cy5‐labeled micelles (500 µL, Cy5‐equivalent concentration of 0.5 µg mL^−1^) were added to the apical side. C) Ex vivo fluorescence imaging was performed on intestinal segments perfused with Cy5‐labeled micelles over a 3‐h period. The intestine segment of SD rats was 8 cm long; the perfusion solution was 3 mL of the OPDEA‐^Cy5^PCL/CEL or PEG‐^Cy5^PCL/CEL solution at a Cy5‐eq. 10 µg mL^−1^. D) Confocal laser scanning microscopy imaging of intestinal cross‐sections and quantification of Cy5 fluorescence intensity along a randomly chosen line from the intestinal cavity to the basolateral side. DAPI staining was applied to the freeze‐sectioned intestine sections. Data are expressed as the mean ± standard deviation from three biological replicates.

For effective oral delivery, the micelles must be capable of penetrating the viscous intestinal mucus layer and achieving efficient uptake by intestinal villi cells^[^ ^28^ ^]^ Thus, the permeability of OPDEA‐^Cy5^PCL/CEL in vitro through the mucus was assessed and quantified by determining their penetration rates through a mucus layer with a thickness of 500 µm (**Figure** **3**A).^[^ ^29^ ^]^ Both OPDEA‐^Cy5^PCL/CEL and PEG‐^Cy5^PCL/CEL micelles were capable of penetrating the mucus layer and reaching the basolateral side, but OPDEA‐^Cy5^PCL/CEL exhibited a significantly faster permeation rate. The calculated permeability coefficients were 3.20 µg h·cm^−2^ for OPDEA‐^Cy5^PCL/CEL and 1.90 µg h·cm^−2^ for PEG‐^Cy5^PCL/CEL. The apparent permeability coefficient (P_app_), which indicates the rate at which the micelles traverse a monolayer Caco‐2 cells on a transwell membrane, was determined to evaluate the transepithelial transport of OPDEA‐^Cy5^PCL/CEL (Figure 3B).^[^ ^30^ ^]^ PEG‐^Cy5^PCL/CEL exhibited minimal transport across the Caco‐2 monolayer, whereas OPDEA‐^Cy5^PCL/CEL progressively traversed to the basolateral side throughout the experimental period, and the concentration of CEL was ≈ 6.6‐fold of PEG‐^Cy5^PCL/CEL at 24 h. The P_app_ value estimated for OPDEA‐^Cy5^PCL/CEL was 2.10 × 10^−6^ cm s^−1^, a significant increase compared to the 3.19 × 10^−7^ cm s^−1^ observed for PEG‐^Cy5^PCL/CEL.

An in situ intestinal perfusion technique was employed to assess the intestinal absorption of OPDEA‐^Cy5^PCL/CEL. In this method, Cy5‐labeled micelles were perfused through a segment of intestine taken from an anesthetized rat. Following the perfusion, the distribution and absorption of the micelles were analyzed through fluorescence imaging (Figure 3C). The intestine perfused with OPDEA‐^Cy5^PCL/CEL exhibited intensive fluorescence, ≈ 10 times greater than that observed with PEG‐^Cy5^PCL/CEL. The intestinal segments were then sectioned and examined by laser confocal microscopy. The intestinal segments perfused with OPDEA‐^Cy5^PCL/CEL displayed stronger fluorescence on the microvillus side (Figure 3D). The intestinal segment perfused with OPDEA‐^Cy5^PCL/CEL exhibited cumulative fluorescence that was three times higher than that observed in segments perfused with PEG‐^Cy5^PCL/CEL, when measured from the lumen to the basal side. The above results indicated that OPDEA‐^Cy5^PCL/CEL had the potential to traverse the inner mucus layer, be internalized by villus cells, and subsequently transport to the basolateral tissue.

### 2.4 Demonstration of Intact Oral Absorption of OPDEA‐PCL FRET Micelles

For micelles to exert their unique biological targeting effects following oral administration, they must maintain structural integrity during gastrointestinal absorption, which is a significant challenge compared to intravenous delivery.^[^ ^18^ ^]^

We first used Cy5‐/Cy5.5‐labeled OPDEA‐PCL FRET micelles to verify the stability of OPDEA‐PCL micelles in the solution and the intestinal medium.^[^ ^31^ ^]^ Within a micelle, most Cy5 and Cy5.5 molecules were sufficiently close and strong FRET occurred, so excitation of Cy5 with 640 nm gave weak Cy5 fluorescence at 670 nm but strong Cy5.5 emission at 720 nm. Once the micelles dissociated, the dye molecules were separated, and the FRET was broken so the FRET peak at 720 nm almost disappeared. As shown in Figure S14 (Supporting Information), the OPDEA‐PCL FRET micelles kept integrated upon dilution with in ddH_2_O, PBS, and 10% FBS, showing minimal dissociation. Notably, FRET signals could be detected below their CMC, suggesting preserved micellar structures even at low concentrations. The micelles also maintained integrity in SGF and SIF, with <5% dissociation observed in SGF (Figure S15, Supporting Information), so most of them should be integrated as micelles in vivo according to above results.

We then investigate the stability of the micelles in the intestinal epithelium cells. The OPDEA‐PCL FRET micelles were added into Caco‐2 cells, and the images and fluorescent spectra were recorded using confocal microscopy. As shown in Figure S16 (Supporting Information), the intracellular FRET signals recorded at different sites at varied times showed strong FRET peak. In the in situ intestinal perfusion model, OPDEA‐PCL FRET micelles were perfused through a segment of intestine (Figure S17, Supporting Information). Following perfusion, the intestinal segments were sectioned and examined by laser confocal microscopy. The FRET signals (720 nm) of micelles in intestinal sections at 3 and 12 h post perfusion demonstrated intestinal absorption of stable micelles. The percentages of FRET micelles were 48.5% at 3 h and 36.7% at 12 h. These findings suggested that the OPDEA‐PCL micelles could be absorbed through the intestine and enter systemic circulation while maintaining their micelle form.

The integrity of the OPDEA‐PCL micelles in the blood circulation was then evaluated. The mouse serum was collected after oral administration with OPDEA‐PCL FRET micelles. As shown in Figure S18 (Supporting Information), the FRET signals (720 nm) of the serum showed a time‐dependent increase from 3–24 h, demonstrating efficient intestinal absorption of intact micelles. Although the Cy5 peak appeared, indicating partial dissociation of the micelles due to shear forces and biomolecular interactions, ≈ 60% FRET micelles remained detectable at 12–24 h. This confirmed both the oral absorption potential and remarkable blood stability of OPDEA‐PCL micelles. The above data indicated that the OPDEA‐PCL/CEL micelle could keep enough integrity to cross the intestinal barrier and entered circulation following oral administration.

### 2.5 OPDEA‐PCL/CEL Micelles Retained in the Gastrointestinal Tract and Effectively Accumulate in the Liver

**Figure 4:**
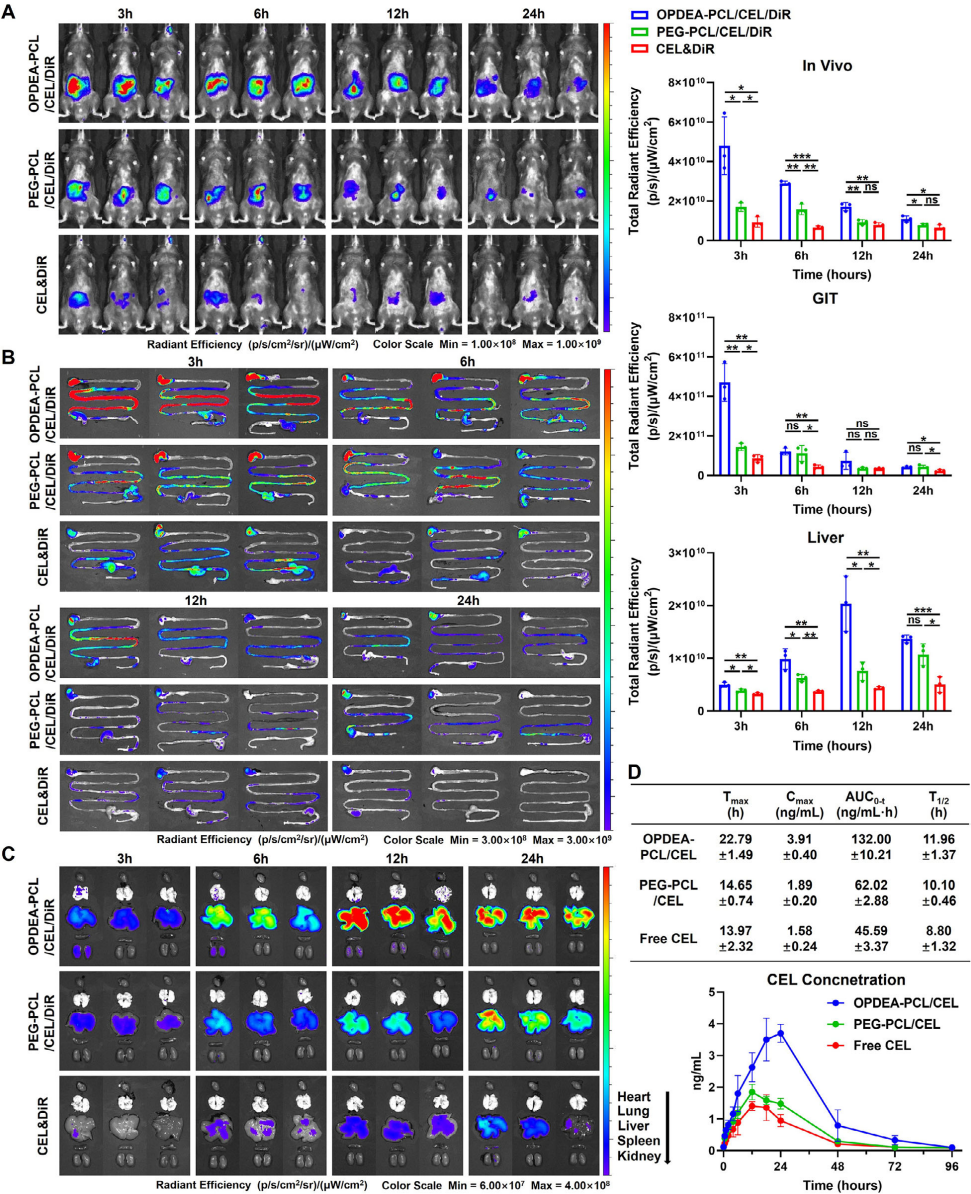
Biodistribution of micelles subsequent to oral administration. A) The in vivo fluorescence imaging and intensity quantification were performed at specific time points following oral administration of DiR‐loaded OPDEA‐PCL/CEL or PEG‐PCL/CEL micelles, or CEL&DiR (DiR‐equivalent dose, 0.1 mg kg^−1^) in C57 mice. B,C) Ex vivo fluorescence imaging was performed, and the fluorescence intensity was measured in the GIT (B) and in major organs, including heart, lungs, liver, spleen, and kidneys (C). D) Plasma CEL concentration during the experimental period and the corresponding pharmacokinetic parameters were plotted following one‐time oral administration of OPDEA‐PCL/CEL, PEG‐PCL/CEL micelles or free CEL. Data are expressed as the mean ± standard deviation from three biological replicates.

The drug delivery systems usually suffer low oral bioavailability due to the rapid emptying effect of the GI tract, so the prolonged retention within the GI tract is crucial for improving drug absorption following oral administration.^[^ ^32^ ^]^ Live imaging showed that 3 h subsequent to oral delivery with OPDEA‐PCL/CEL/DiR, intense fluorescence was detected in the upper abdomen of mice (**Figure** **4**A), reached a maximum at 6 h, and decreased over time. However, it could still be detected at 24 h post administration, while the fluorescence intensity in the abdomen of the free DIR or PEG‐PCL/CEL/DiR treated mice was much lower and hardly detectable at 24 h. Ex vivo imaging showed that strong fluorescence was detected in the GIT subsequent to oral delivery with OPDEA‐PCL/CEL/DiR (Figure 4B). However, the fluorescence intensity in the GIT of the free DIR and PEG‐PCL/CEL/DiR groups decreased rapidly over time. This indicated that the OPEDA‐PCL micelles could be retained in the GI tract, providing a more favorable opportunity for sufficient drug absorption. The fluorescence intensities of the organs were consistent with the above results, showing that OPDEA‐PCL/CEL/DiR accumulated much more in the liver (Figure 4C; Figure S19, Supporting Information) than the other organs.

The pharmacokinetic profile of OPDEA‐PCL/CEL micelles was then evaluated. Following oral administration of OPDEA‐PCL/CEL, plasma concentrations of CEL were observed to rise over the initial 24‐h period (Figure 4D). In contrast, oral dosing with free CEL or PEG‐PCL/CEL micelles resulted in significantly lower plasma levels at every time point. OPDEA‐PCL/CEL micelles had an AUC_0–t_ value 2.90 or 2.13‐fold of free CEL or PEG‐PCL/CEL micelles. The larger T_1/2_ of OPDEA‐PCL/CEL micelles implied a longer circulation time in the bloodstream. These results indicate that OPDEA‐PCL/CEL micelles markedly improved the oral bioavailability of CEL.

The uptake of OPDEA‐PCL/CEL micelles by the main cell populations (hepatocytes and Kupffer cells) in the liver was analyzed by flow cytometry (Figure S20, Supporting Information). At 12 h after oral administration, the overall Cy5 positive rate increased by 22.5% in the hepatocytes, and increased by 68.3% in Kupffer cells. Immunofluorescence staining of liver cryosections yielded similar results (Figure S21, Supporting Information).

### 2.6 OPDEA‐PCL/CEL Micelles Formed an HDL Corona and Actively Targeted the Liver

**Figure 5:**
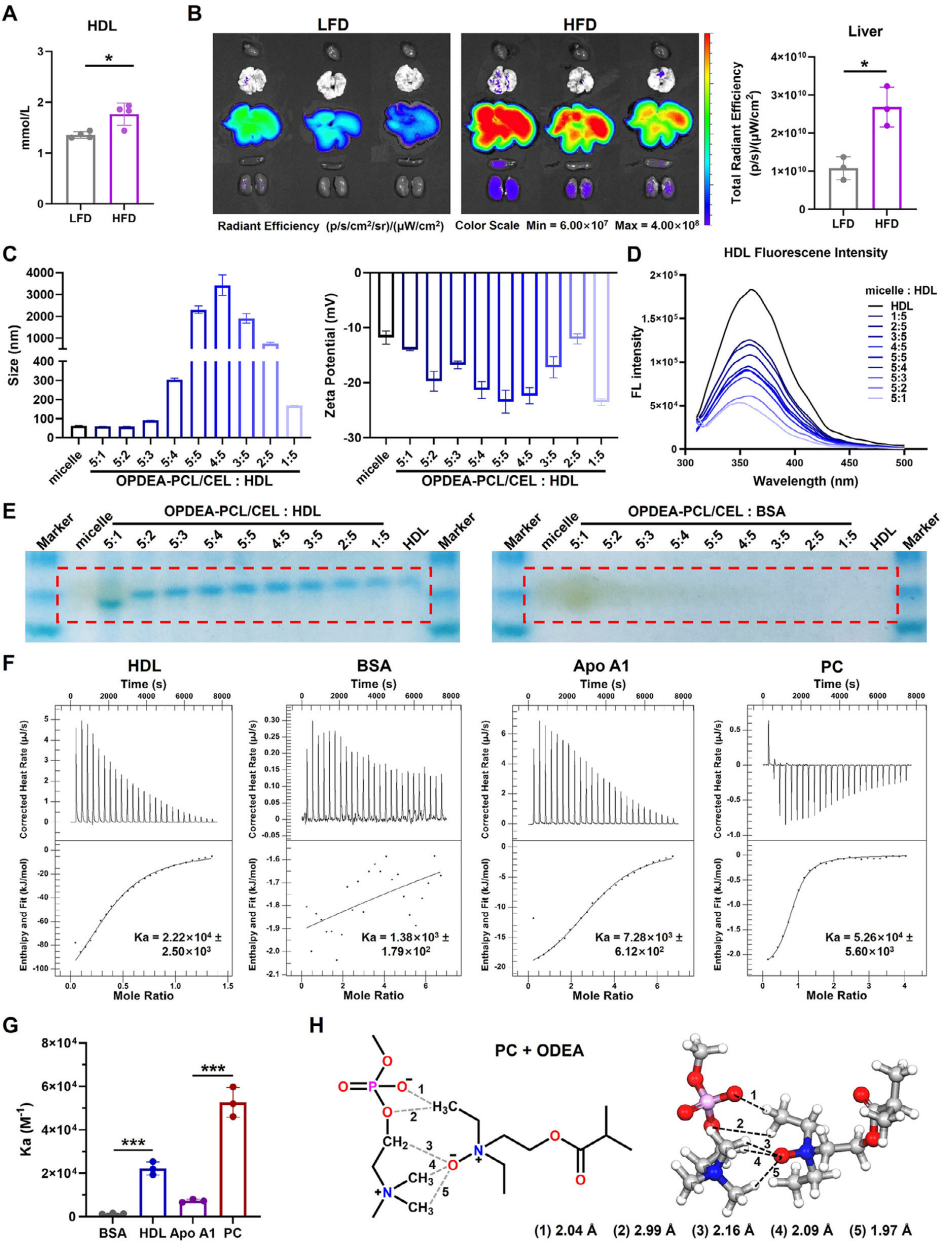
Liver‐targeted mechanism studies of OPDEA‐PCL/CEL micelles. A) HDL contents in the plasma of C57 mice fed with LFD or HFD (*n* = 4). B) Ex vivo fluorescence imaging and intensity quantification in the liver of DiR‐loaded OPDEA‐PCL/CEL micelles (DiR‐eq. 0.1 mg kg^−1^) after oral administration for 12 h in C57 mice fed with LFD or HFD (*n* = 3). C) OPDEA‐PCL/CEL micelle changes in size and zeta potential measured by DLS after mixing with HDL at different mass ratios (*n* = 3). D) Fluorescence quenching curves of HDL protein after incubating with micelles at different mass ratios detected by fluorescence spectrometer. E) Native PAGE electrophoresis of HDL or BSA after mixing with OPDEA‐PCL/CEL micelles at different mass ratios. The red square on the left showed OPDEA‐PCL/CEL micelles retarded HDL motion, while the right square showed that OPDEA‐PCL/CEL micelles had no effect on BSA. See Figure S19 (Supporting Information) for details. F,G) The Isothermal Titration microcalorimetry (ITC) curves (F) and the calculated binding constant (K_a_) (G) of HDL or BSA with OPDEA‐PCL/CEL micelles (OPDEA‐PCL‐eq. 1 mg mL^−1^, 37 °C) (*n* = 3). H) Binding‐energy simulation of an OPDEA unit with the phosphatidylcholine (PC) ionic moiety by Molecular Dynamics (Materials Studio software version 19.1.0.2353). Data are expressed as the mean ± standard deviation from biological replicates.

The complexity of protein corona in influencing the transformation and transport of micelles, as well as in inducing their biological functional effects both in vitro and in vivo, has been demonstrated.^[^ ^33^ ^]^ Lipoproteins are highly abundant in plasma.^[^ ^34^ ^]^ The composition of high‐density lipoprotein (HDL) in the protein corona on the surface of micelles in the bloodstream significantly impacts their biological functions.^[^ ^21^ ^]^ Furthermore, the HDL protein content and other blood lipid indices in the plasma of MASLD mice were elevated (**Figure** **5**A; Figure S22, Supporting Information). Ex vivo imaging suggested that after oral administration, OPDEA‐^Cy5^PCL/CEL accumulated more in the livers of the mice with HFD‐induced MASLD than the normal mice (Figure 5B; Figure S23, Supporting Information). So HDL may play an important role in the enhanced accumulation in the MASLD livers.

The interaction of the micelles with HDL was then investigated. As seen from Figure 5C and Figure S24 (Supporting Information), the presence of HDL significantly changed the particle sizes and zeta potentials of OPDEA‐PCL/CEL micelles and even caused micelle aggregation to several micrometers. We then conducted the protein intrinsic fluorescence quenching method to detect interactions between OPDEA‐PCL/CEL micelles and HDL. A fluorescence quenching effect (31.6%) was observed even at a low micelle‐to‐HDL mass ratio (1:5). As the concentration of OPDEA‐PCL/CEL micelles increased, the HDL fluorescence was gradually quenched, reaching 70.8% quenched at 5:1 mass ratio (Figure 5D). Native‐PAGE analysis further demonstrated that as the ratio of HDL to OPDEA‐PCL/CEL increased, the complex mobility decreased. BSA, a representative serum protein, showed no interaction with the OPDEA‐PCL/CEL micelles, indicating the specific HDL binding feature of OPDEA‐PCL (Figure 5E; Figure S25, Supporting Information). Isothermal titration calorimetry assay confirmed a strong interaction between OPDEA‐PCL/CEL micelles and HDL, with a binding constant K_a_ of 2.22 × 10^4^, much higher than that for BSA (K_a_ = 1.38 × 10^3^). The major lipid component of HDL is phospholipids, and the main protein component is apolipoprotein Apo‐A1.^[^ ^23^ ^]^ Titration of OPDEA‐PCL/CEL micelles with the separate components showed a weaker interaction with Apo‐A1 (K_a_ = 7.28 × 10^3^) but a stronger interaction with phospholipids (K_a_ = 5.26 × 10^4^) (Figure 5F,G). Molecular dynamics simulations suggested hydrogen bond formation between the ionic head of phospholipid and the N^+^‐O^−^ structure in OPDEA (Figure 5H). Based on these results, we hypothesized that the phospholipid‐binding ability of OPDEA made the micelles adsorb HDL to form a protein corona. As the affinity of OPDEA‐PCL/CEL micelles to Apo‐A1 compartment was only one tenth of phospholipid, one may image that HDL bound onto the micelles via its phospholipid side, exposing the SR‐B1 receptor for liver targeting.

### 2.7 OPDEA‐PCL/CEL Micelles Ameliorated Metabolic Dysfunctions in Mouse MASLD Model

**Figure 6:**
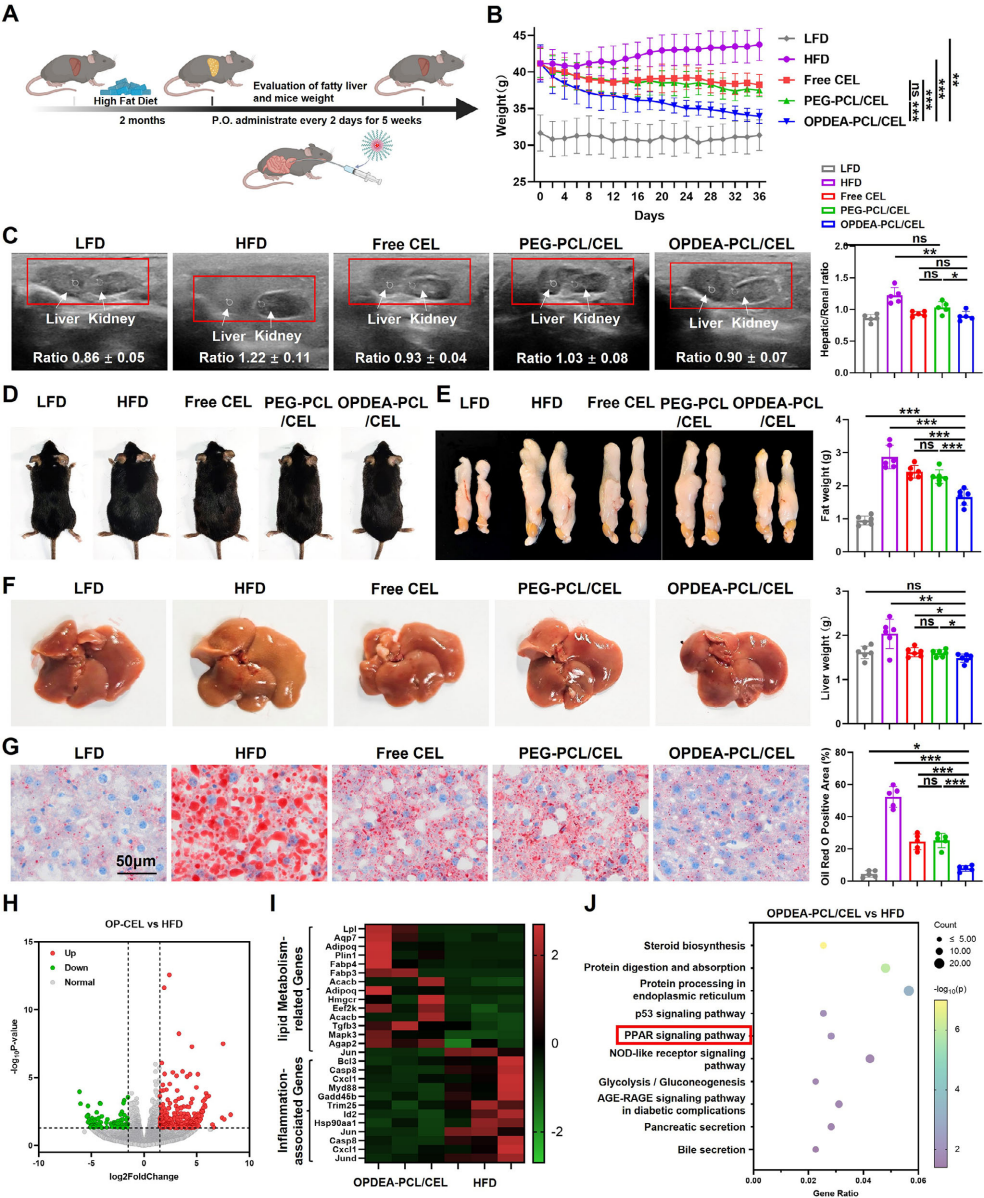
The therapeutic efficacy and mechanism study of OPDEA‐PCL/CEL micelles in the mouse MASLD model. A) Treatment schedule of OPDEA‐PCL/CEL micelles. C57 mice were fed with HFD for 2 months to establish the MASLD model; then the mice were treated with ddH_2_O, free CEL, PEG‐PCL/CEL, or OPDEA‐PCL/CEL (CEL‐eq. 2 mg kg^−1^) by oral administration every two days for five weeks. B) The average body weight curves of each gouup (*n* = 6). C) Ultrasound evaluation and quantification of the hepatic/renal ratios in mice after treatments (*n* = 5). D) Representative images of the mice following oral administration in each group. E) Images and weights of abdominal fat tissues of each group (*n* = 6). F) Liver images and weights (*n* = 6). G) The images of Oil Red O stained liver sections and semiquantitative analysis of lipid droplet area across five randomly selected fields by ImageJ software (version 1.52i). H–J) The volcano plot (H), heatmap (I) of differentially expressed genes (DEGs) and KEGG enrichment analysis (J) between OPDEA‐PCL/CEL and HFD group livers by RNA sequencing (*n* = 3). Data are expressed as the mean ± standard deviation from biological replicates.

The MASLD model in mice was induced by feeding C57BL/6J mice a high‐fat diet (HFD, 60 kcal % fat). MASLD mice received oral treatments with free CEL, PEG‐PCL/CEL micelles or OPDEA‐PCL/CEL micelles (CEL‐eq. 2 mg kg^−1^) every 2 days for 5 weeks, during which their body weights were monitored (**Figure** **6**A). During the treatment period, the average body weight of HFD‐fed mice steadily increased, while it remained stable of those fed with LFD. In the OPDEA‐PCL/CEL treatment group, the average body weight gradually decreased, ≈ 22.4% reduction compared to the HFD group at Day 36, and reached to that of LFD group. The free CEL or PEG‐PCL/CEL micelle treatment groups slightly reduced the body weight, 12.5% or 14.2% reductions, respectively (Figure 6B). During the treatment period, ultrasound scanning was employed to assess the lipid accumulation in livers. The hepatorenal index, defined as the ratio of the echogenicity of the liver to that of the renal cortex, is a semiquantitative ultrasonography method to estimate steatosis in the clinic. It was used here to confine above results (Figure 6C). Upon completion of the experiments, the mice on a normal diet exhibited leaner shape, greater agility, and glossy black fur, whereas HFD‐fed mice displayed body extension, dull fur, and reduced activity. However, mice in the OPDEA‐PCL/CEL group showed marked improvements in body condition and fur quality, close to the LFD group. In contrast, the free CEL and PEG‐PCL/CEL groups exhibited modest improvements (Figure 6D).

Abdominal fat content serves as an indicator of lipid metabolism. As shown in Figure 6E, the HFD group displayed significant abdominal fat accumulation. The abdominal fat mass was reduced by 15.6%, 20.6%, or 42.3% in the free CEL, PEG‐PCL/CEL, and OPDEA‐PCL/CEL groups, respectively. LFD group mice had healthy reddish livers; the livers of HFD‐fed mice appeared yellowish‐brown with granular lipid deposits and larger sizes. After oral administration of OPDEA‐PCL/CEL, the liver quality was significantly normalized. The livers of free CEL and PEG‐PCL/CEL treated mice showed mild improvement, but still retained scattered lipid granules (Figure 6F). Oil Red O staining, an indicator of hepatic fat deposition, was then performed on frozen liver sections of each group (Figure 6G). In the LFD group, minimal lipid droplets were found in the hepatocytes, with less than 5% of the total area stained red. In contrast, HFD group hepatocytes had large lipid droplets repelling the cell nuclei to the periphery, with over 50% of the total stained red. Treatment with OPDEA‐PCL/CEL resulted in a marked reduction of lipid droplets, with only 8% of the area stained red, close to normal levels. The free CEL and PEG‐PCL/CEL groups had 24.4% and 25.2% of the total area stained red, respectively, indicating relative less efficacious modulation.

Subsequently, serum biochemical markers were measured to evaluate liver function (Figure S26, Supporting Information). Treatment with free CEL, PEG‐PCL/CEL and OPDEA‐PCL/CEL markedly decreased the levels of TC and LDL in serum, with OPDEA‐PCL/CEL micelles achieving the most pronounced reduction. OPDEA‐PCL/CEL micelles effectively also normalized the serum levels of AST and ALT in MASLD mice, comparable to or even better than free CEL, PEG‐PCL/CEL treated mice. LC‐MS analysis was conducted simultaneously to measure CEL content in serum and liver tissue (Figure S27, Supporting Information). The serum CEL content of OPDEA‐PCL/CEL group was 1.9 times or 2.1 times higher than that of free CEL and PEG‐PCL/CEL groups, and the liver CEL content was 2.3 times and 2.0 times higher, respectively. Transcriptomic analysis of liver tissues revealed that OPDEA‐PCL/CEL treatment significantly promoted lipid metabolism while attenuating inflammatory responses (Figure 6H,I), further confirmed the activation of PPAR signaling pathway by enrichment analysis (Figure 6J).

### 2.8 OPDEA‐PCL/CEL Micelles Reduced Systemic Toxicity of CEL

**Figure 7:**
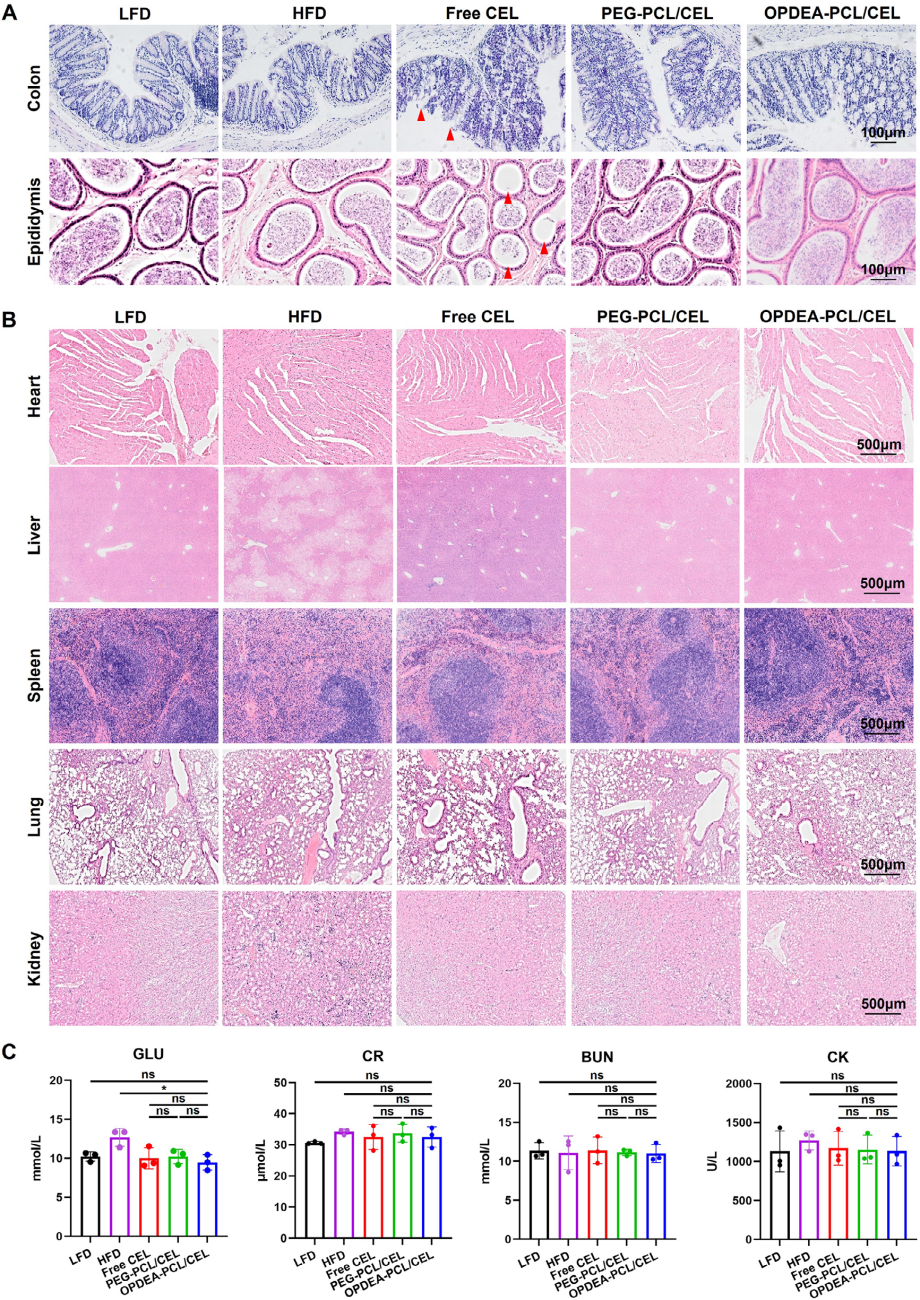
Biosafety assessments in the MASLD mice at the end of the treatments are shown in Figure 6. A) H&E staining of colon and epididymis in mice following oral administration (The red arrows indicate the tissue damage). B) H&E staining of other organs in mice following oral administration. (C) Serum biochemistry analysis of glucose (GLU), creatinine (CR), blood urea nitrogen (BUN), and creatine kinase (CK) levels in mice after oral administration (*n* = 3). Data are expressed as the mean ± standard deviation from biological replicates.

The biosafety of free CEL, PEG‐PCL/CEL, or OPDEA‐PCL/CEL was assessed through histological examinations of major organs and serum biochemical analyses after five weeks of oral treatments in MASLD C57 mice. H&E staining of colon sections showed that free CEL disrupted the microvillus structure and induced inflammation (**Figure** **7**A). Epididymal section staining showed that free CEL reduced the number of spermatozoa in the epididymal lumen, indicating reproductive toxicity. Conversely, no obvious such toxicity was observed in these tissues after PEG‐PCL/CEL or OPDEA‐PCL/CEL treatments (Figure 7A). No pathological damage was detected in heart, spleen, lungs, or kidneys among all treatment groups (Figure 7B). The livers of LFD mice exhibited tightly arranged hepatocytes with integrated nuclei and clear lobular structures, while the HFD mice showed prominent lipid droplets and irregular hepatocytes. Treatment with free CEL and PEG‐PCL/CEL resulted in partial reduction of lipid vacuoles, while OPDEA‐PCL/CEL treatment led to the most decrease in vacuole formation, with intact liver structure and normalized hepatocyte morphology, close to that of LFD normal mice (Figure 7B). Serum biochemical analyses showed no significant deviation in cardiac injury markers or renal function indicators between the treatment and LFD groups (Figure 7C). To further evaluate systemic toxicity, normal mice were treated for three weeks. Complete blood counts revealed no significant changes in red blood cell counts or hemoglobin levels in the OPDEA‐PCL/CEL and PEG‐PCL/CEL groups, with a slight decrease in the free CEL group (Figure S28, Supporting Information). Platelet levels remained stable amongst all groups. Subsequently, serum biochemistry tests were conducted to evaluate hepatorenal functions and cardiac injury markers, with no significant differences observed (Figure S29, Supporting Information). Overall, these findings suggested that OPDEA‐PCL/CEL micelles had improved biocompatibility and reduced systemic side effects.

### 2.9 OPDEA‐PCL/CEL Micelles Exhibited Superior Therapeutic Efficacy over Simvastatin in Mouse MASLD Model

**Figure 8:**
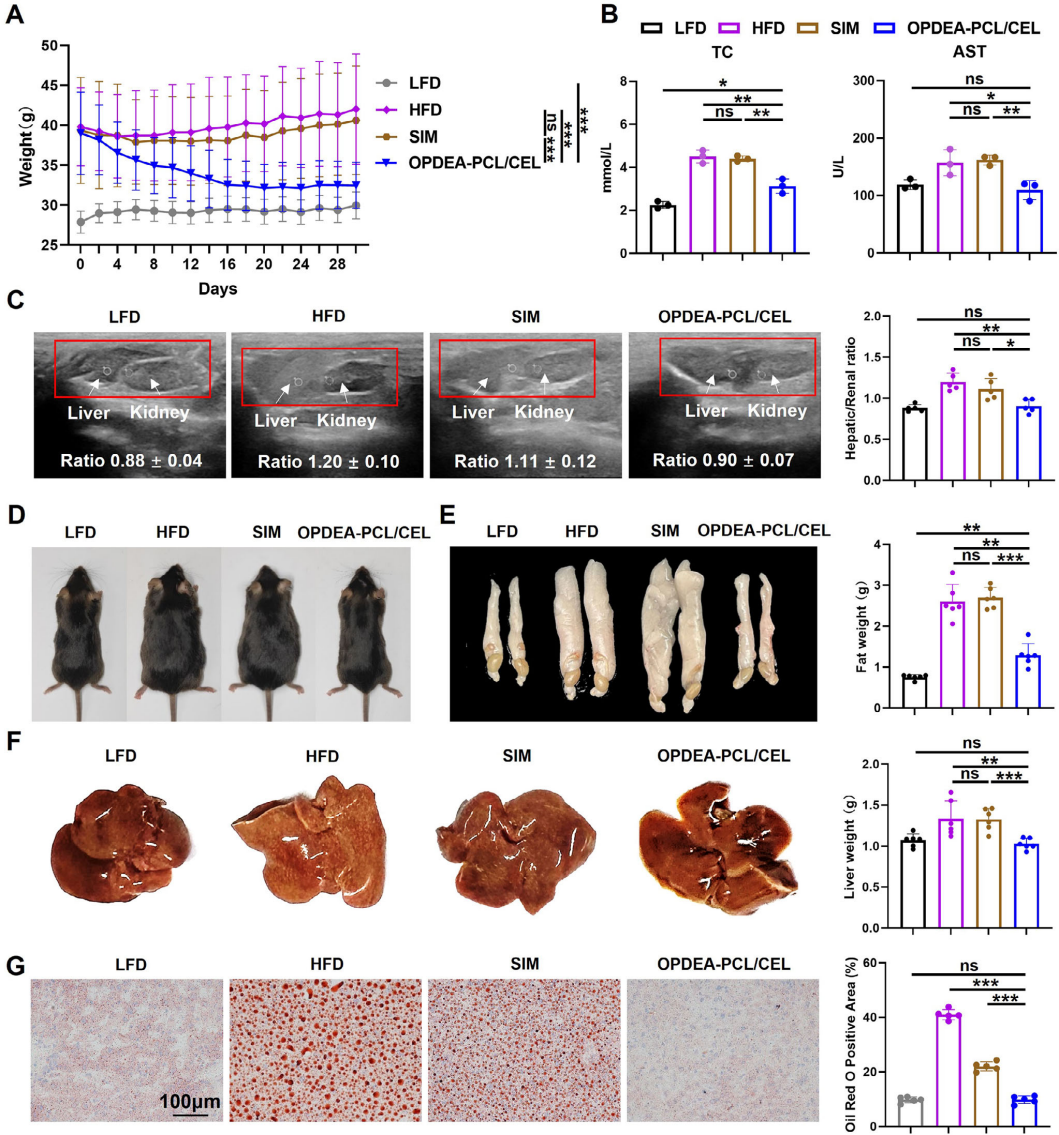
Comparison of the therapeutic efficacy of OPDEA‐PCL/CEL micelles with SIM in the mouse MASLD model. The mice were treated with ddH_2_O, SIM (20 mg kg^−1^) and OPDEA‐PCL/CEL (CEL‐eq. 2 mg kg^−1^) by oral administration every two days for four weeks. A) Body weight curves (*n* = 6). B) Serum biochemistry analysis of TC and AST levels in mice after oral administration (*n* = 3). C) Ultrasound evaluation and quantification of the hepatorenal ratios in mice after treatments (*n* = 5). D) Representative images of mice after oral administration. E) Images and weights quantification of abdominal fat tissues (*n* = 6). F) Liver morphology and weights quantification (*n* = 6). G) Oil Red O staining of liver sections and semiquantitative analysis of lipid droplet area across five randomly selected fields by ImageJ software (version 1.52i). Data are expressed as the mean ± standard deviation from three biological replicates.

The therapeutic efficacy of OPDEA‐PCL/CEL micelles was also compared with simvastatin (SIM), a clinically used antihyperlipidemic agent.^[^ ^35^ ^]^ MASLD mice were orally administered with OPDEA‐PCL/CEL micelles (CEL‐eq. 2 mg kg^−1^) and SIM (20 mg kg^−1^)^[^ ^36^ ^]^ every two days for four weeks. Similarly, the average body weight gradually decreased by 22.4% after OPDEA‐PCL/CEL treatment, approaching the levels of LFD group. However, the simvastatin‐treated group did not exhibit significant reduction in body weight (**Figure** **8**A). Serum biochemical analysis showed that OPDEA‐PCL/CEL treatment lowered total cholesterol, ALT, and AST levels, and decreased blood glucose levels, while simvastatin had no significant effects (Figure 8B; Figure S30, Supporting Information). Hepatorenal ratios demonstrated a more pronounced improvement in the OPDEA‐PCL/CEL group compared to the simvastatin group assessed by ultrasound (Figure 8C). Comparisons of body shapes and abdominal fat content further suggested that simvastatin was relatively less effective than OPDEA‐PCL/CEL micelles (Figure 8D,E). Liver morphology was normalized after OPDEA‐PCL/CEL treatment, whereas there were still some visible yellowish lipid granules in simvastatin group (Figure 8F). Oil Red O staining of liver sections further revealed the lipid area decreased from 41.0% to 9.8% after OPDEA‐PCL/CEL micelles treatment, with the stained area lessened to 22.0% in the simvastatin group (Figure 8G).

## 3 Conclusion

In conclusion, this study established OPDEA‐PCL as an effective platform for the oral delivery of CEL in the treatment of MASLD. OPDEA‐PCL effectively encapsulated CEL with excellent stability. These micelles were able to tolerate the harsh acidic conditions of the gastrointestinal tract, enabling efficient absorption across the intestinal mucosa through transcytosis. Subsequently, upon entering the bloodstream, the OPDEA‐PCL/CEL micelles specifically captured HDL, forming a functional protein corona that facilitated active liver targeting and CEL accumulation. The resultant significant reduction of hepatic lipid and mitigation of liver steatosis were superior to those of free CEL, PEG‐PCL/CEL micelles and clinical used simvastatin, endowing it as a safe and effective oral treatment of MASLD.

## 4 Experimental Section

### Statistical Analysis

Results are presented as means ± SD. Group comparisons were made using an unpaired two‐tailed t‐test. Statistical significance: ns, no significance; ^*^, *p* < 0.05; ^**^, *p* < 0.01; ^***^, *p* < 0.001.

## Conflict of Interest

The authors declare no conflict of interest.

## Author Contributions

C.X., H.Z., K.W., and H.H. contributed equally to this work. This research was funded by Y.S., N.Q., and X.X. N.Q., Y.S., and X.X. are corresponding authors. C.X., Y.S., N.Q., and X.X. designed the research. N.Q. and X.X. supervised the research. C.X., J.Z., Y.L. synthesized the polymers. C.X., H.Z., H.H., D.X, executed experiments. C.X., H.Z., X.W., K.W., and J.C. analyzed the data. C.X. and N.Q. wrote the manuscript, N.Q., Y.S., and X.X. revised the manuscript and all authors discussed and commented on it.

## Supporting information



Supporting Information

## Data Availability

The data that support the findings of this study are available in the supplementary information of this article.
