# Supplementary material for: Oral Celastrol Micelles Forming High‐Density Lipoprotein Corona Targeting Hepatocytes for MASLD Treatment
Source: Adv Sci (Weinh). 2025 Sep 23;12(45):e00854. doi: 10.1002/advs.202500854 (PMC12677692; doi:10.1002/advs.202500854)
Supplement: Supplementary file 1 — Supporting Information [file ADVS-12-e00854-s001.docx]

Supporting Information

Oral Celastrol Micelles Forming High-Density Lipoprotein Corona Targeting Hepatocytes for MASLD Treatment

Chang Xu, Haoru Zhu, Kai Wang, Haitao Hu, Xuyong Wei, Dongdong Xu, Jing Zhang, Yanpeng Liu, Jun Chen, Youqing Shen*, Nasha Qiu*, Xiao Xu*

C. Xu

Hangzhou First People’s Hospital, Zhejiang University School of Medicine, Hangzhou 310058, China.

E-mail: shenyq@zju.edu.cn; qiunasha@zju.edu.cn; zjxu@zju.edu.cn.

C. Xu, H. Hu, X. Wei, D. Xu, Y Liu, N Qiu

Key Laboratory of Integrated Oncology and Intelligent Medicine of Zhejiang Province, Affiliated Hangzhou First People’s Hospital, School of Medicine, Westlake University, Hangzhou 310006, China.

H. Zhu, J. Zhang, Y. Shen

Key Laboratory of Smart Biomaterials of Zhejiang Province and Key Laboratory of Biomass Chemical Engineering of the Ministry of Education of China, College of Chemical and Biological Engineering, Zhejiang University, Hangzhou 310058, China.

K. Wang, J. Chen

School of Clinical Medicine, Hangzhou Medical College, Hangzhou 310053, China.

C. Xu, X. Xu

Zhejiang University School of Medicine, Hangzhou 310058, China.

X. Xu

Hepatobiliary Center, The First Affiliated Hospital of Nanjing Medical University, Nanjing 210000, China.

Keywords: MASLD, HDL corona, celastrol, liver-targeting micelles

**Methods and Materials**

**1. Materials**

Celastrol (CEL) (purity ≥ 98%, as determined by HPLC) was procured from Tauto Biotech (Shanghai, China). Methanol, acetonitrile, trichloromethane, sucrose, paraformaldehyde (PFA), and other commonly used chemical reagents were sourced from Sinopharm Chemical Reagent Co., Ltd. (Shanghai, China). LysoTracker Red DND-99, Hoechst 33342 and ProLong Gold Antifade Mountant with DNA Stain DAPI were procured from Thermo Fisher Scientific (Invitrogen, Eugene, Oregon, USA). Mito-Tracker Green and the apoptosis assay kit was procured from Beyotime Biotechnology (Shanghai, China). O.C.T. Compound was obtained from Tissue-Tek (SAKURA, USA). DiR (1,1'- dioctadecyl-3,3,3',3'-tetramethylindotricarbocyanine iodide) was purchased from AAT Bioquest, Inc. (California, USA). Antibodies and related reagents used in this study were detailed in **Table S1**.

**2. Cell lines and animals**

AML12 (mouse hepatocytes), HepG2 (human hepatocellular carcinoma) and Caco-2 (human epithelial colorectal adenocarcinoma) cell lines were sourced from the Cell Bank of the Chinese Academy of Sciences. The AML12 cell was cultured in a nutrient-rich 1640 medium (containing 10% FBS, 1% (v/v) penicillin-streptomycin, 1% (v/v) Insulin-Transferrin-Selenium and 40 ng/mL Dexamethasone). HepG2 cells were cultured in a nutritious high glucose DMEM medium (containing 10% FBS and 1% (v/v) penicillin-streptomycin). Caco-2 cells were cultured in a nutritious MEM medium (containing 20% FBS, 1% (v/v) penicillin-streptomycin and 1% (v/v) non-essential amino acid). All cells were cultured in cell incubators at 37 ℃ with 5% CO_2_.

C57BL/6 mice (male, 6-8 weeks) and Sprague-Dawley (SD) rats (male, 250-300 g) were sourced from Zhejiang Center of Laboratory Animals (Hangzhou, China). Mice and rats were maintained in a thermostatic environment (22 ± 2 °C) with a 12-h day-night cycle in the Zhejiang Center of Laboratory Animals under SPF environment. Approval for all animal experiments was obtained from the Institutional Animal Care and Use Committee of Zhejiang Academy of Medical Sciences (Approval No. ZJCLA-IACUC-20010214).

The MASLD model in mice was induced by feeding four-week-old male C57BL/6 mice with HFD containing 60% kcal from fat (Research Diet, D12492) for a total of 8~12 weeks to induce MASLD.^[1]^ Control mice were fed with a standard chow diet during the same period. Mice were included in the experiment once their body weight exceeded 40 grams. Throughout the feeding period, the body weight and food intake were recorded weekly to assess overall health and ensure appropriate diet consumption.

**3. Synthesis of poly[2-(N-oxide-N,N-diethylamino)ethyl methacrylate]-poly(ε-caprolactone) (OPDEA-PCL)**

The block copolymers (OPDEA to PCL at 2:5, 3:5, 4:5, 5:5, 6:5, and 8:5) were synthesized according to the reported methods^[2]^, see Supplementary Information Fig. S1~S6.

**4. Liquid Chromatography-Mass Spectrometry (LC-MS) Analysis of CEL**

LC-MS analysis of CEL was performed using an Agilent 1290 Infinity II UHPLC system coupled to a 6495 Triple Quadrupole mass spectrometer (Agilent Technologies, USA). Chromatographic separation was performed on a ZORBAX Eclipse XDB-C18 column (RRHD, 2.1 × 100mm, 1.8-Micron; Agilent Technologies). The mobile phase was consisted of 0.1% formic acid in water (A) and 0.1% formic acid in methanol (B). A gradient elution was applied starting at 20% B, which was linearly increased to 90% B over 8 min, followed by holding at 90% B for 10 min. The flow rate was adjusted to 0.2 mL/min, with the column temperature kept at 40°C. The volume of sample injected was 4 μL.

Mass spectrometry was performed using an Agilent Jet Stream electrospray ionization (AJS ESI) source in positive ion mode. The ion source parameters were set as follows: the capillary voltage was 3000 V, with the nozzle voltage set to 1500 V. The drying gas temperature was 270℃, with a drying gas flow rate of 14 L/min, and the nebulizer pressure was maintained at 25 psi. The sheath gas flow rate was 11 L/min with a temperature of 350℃. The gas curtain was maintained using high-purity nitrogen. Precursor-to-product ion transitions were monitored in multiple reaction monitoring (MRM) mode for quantification. The optimized mass spectrometer settings for CEL involved the transition from m/z 451.2 (precursor ion) to m/z 201.1 (product ion) with a collision energy of 35 eV and a cell accelerator voltage (CAV) of 5 V. Data acquisition and processing were carried out using MassHunter Workstation software (Agilent Technologies).

**5. Preparation and characterization of micelles**

To prepare the micelles, OPDEA-PCL and CEL were initially dissolved in a 5 mL volume of dichloromethane. Following solvent evaporation at 40℃, the resultant film was rehydrated with 3 mL of deionized water while stirring at 300 rpm for 1 h. The dispersion was subsequently homogenized using an ultrasonic homogenizer set at 250 W for 3 min. Purification was achieved through dialysis for 2 h using a dialysis bag (25 mm diameter, MWCO 3500), yielding the purified OPDEA-PCL/CEL micelles. The PEG-PCL/CEL micelles were fabricated following an identical protocol. Subsequently, the OPDEA-^Cy5^PCL/CEL, OPDEA-PCL/CEL/Dir, PEG-^Cy5^PCL/CEL and PEG-PCL/CEL/Dir micelles were also prepared using the same methodology. The particle sizes and zeta potentials of CEL-loaded micelles in HEPES buffer (10 mM, pH 7.4) were characterized using dynamic light scattering (DLS, Zetasizer Nano ZS90, Malvern Instruments, UK). The encapsulation efficiency (EE) and drug loading content (DL) of micelles were calculated from the amount of CEL loaded in the micelles and analyzed using the LC-MS as mentioned above.

Then EE and DL were calculated by the following equations:

EE (%) = (W_E_ / W_CEL_) ×100%

DL (%) = W_E_ / (W_CEL_+W_M_) ×100%

W_E_: weight of CEL encapsulated in micelles; W_CEL_: weight of total CEL added; W_M_: weight of blank micelles.

**6. Critical micelle concentration (CMC) determination**

Nile red was dissolved in CH_2_Cl_2_ and evaporated. Then 0.5 mL of OPDEA-PCL or PEG-PCL at varied concentrations (0.5 to 500 μg/mL) were added and stirred in the dark for 12 h. Fluorescence intensity was measured at excitation and emission wavelengths of 543 nm and 620 nm, using a microplate reader (SpectraMax iD5, Molecular Devices, USA). The CMC was determined by identifying the intersection point of two linear regression lines derived from plots of fluorescence intensity vs. micelles concentration.

**7. *In vitro* stability of micelles**

The *in vitro* stability of OPDEA-PCL/CEL and PEG-PCL/CEL micelles in various aqueous and biorelevant media was investigated by monitoring particle sizes and related PDI. Briefly, The micelles solution (1 mL, polymer-eq. dose, 1 mg/mL) was added to 4 mL of deionized water, PBS (pH 7.4), simulated intestine fluid (SIF) , or simulated gastric fluid (SGF) prepared as reported. The micelle dispersions were then incubated at 37 ℃ under shaking at 100 rpm. The micelles were stored at room temperature for a long-term stability study. The particle size and polydispersity index (PDI) of the micelles were measured using dynamic light scattering (DLS) at predetermined time intervals.

**8. *In vitro* profile release of CEL from micelles**

CEL-loaded micelles (1 mg/mL, 1 mL) were placed into a dialysis bag (MWCO 3500) and submerged in 20 mL of release medium, which consisted of PBS (pH 7.4), simulated intestinal fluid (SIF), or simulated gastric fluid (SGF) containing 0.5% Tween 80. The system was kept at 37℃ and stirred continuously at 100 rpm via a water-bath shaker. At specified time points, a certain volume (0.5 mL) of the sample was withdrawn and replaced with an equivalent volume of fresh medium. The concentration of released CEL was quantified by LC-MS, as previously described.

**9. Assessment of cellular viability**

The cytotoxic effects of free CEL, PEG-PCL/CEL micelles or OPDEA-PCL/CEL micelles on AML12 and HepG2 cells were evaluated using the CCK8 assay. In brief, AML12 cells or HepG2 cells were seeded at a density of 5×10^3^ cells per well in 96-well plates and subsequently treated with free CEL, PEG-PCL/CEL micelles or OPDEA-PCL/CEL micelles at various concentrations for 48 h. After discarding the cell culture medium, the cells were incubated with CCK8 kit for 2 h. Absorbance was measured at 450 nm using a microplate reader (SpectraMax iD5, Molecular Devices, USA). Cell viability was presented as a percentage compared to the untreated control group.

**10. Cell apoptosis analysis**

AML12 cells were plated in 6-well plates at a density of 2×10^5^ cells per well and incubated overnight to allow for cell attachment. Subsequently, the cells were treated with free CEL, OPDEA-PCL/CEL micelles, or PEG-PCL/CEL micelles at CEL-eq. concentrations from 0 to 10 μg/mL. After 24 h, the cells were washed three times with PBS and stained with Propidium Iodide and APC Annexin V using the Annexin V-APC/PI Apoptosis Kit (Multi Science, China). Apoptosis was assessed using BD FACSCanto II flow cytometry (BD Biosciences, USA), with subsequent data analysis performed using FlowJo software (Version 10).

**11. Cellular uptake and mitochondrial colocalization**

AML12 cells (5×10^5^ cells/well) were seeded in 6-well plates and incubated overnight with OPDEA-^Cy5^PCL/CEL micelles or PEG-^Cy5^PCL/CEL micelles (the Cy5-equivalent dose was 0.5 μg/mL). After incubation, the cells were washed with PBS and analyzed using BD FACSCanto II flow cytometry (BD Biosciences, USA), and the cellular uptake rates were quantified using FlowJo software (v10).

For subcellular distribution tracking, AML12 cells (1×10^5^ cells/well) were seeded in glass-bottom culture dishes and incubated overnight with OPDEA-^Cy5^PCL/CEL micelles or PEG-^Cy5^PCL/CEL micelles (Cy5-eq. dose, 0.5 μg/mL). After 6 h, the nuclei were stained with Hoechst 33342 (2 μM, Invitrogen) and mitochondria was stained with MitoTracker Green (200 nM, Invitrogen) for 15 min. The cells were then washed three times with PBS and imaged using an Olympus FV3000 confocal microscope (Olympus, Japan). Pearson's correlation coefficients were determined using ImageJ software (version 1.52i) by analyzing three randomly selected lines within the LSCM.

**12. Oil Red O staining**

AML12 cells were plated in 6-well plates at a density of 2×10^5^ cells per well and incubated overnight to allow for cell attachment. Subsequently, the cells were incubated with oleate (OA, 0.5 mM) and treated with free CEL, OPDEA-PCL/CEL micelles, or PEG-PCL/CEL micelles at CEL-equivalent concentration of 0.5 μg/mL for 48 h. After fixation with 4% paraformaldehyde for 15 minutes at RT, the cells were rinsed three times with PBS and subsequently stained with a freshly prepared Oil Red O working solution (Oil Red O Staining Kit, Beyotime, China) for 15 min at RT. Unwanted stain was eliminated by gently rinsing the cells with 60% isopropanol. Subsequently, the cells were washed with distilled water and visualized using an Olympus CKX53 microscope (Olympus, Japan). The Oil Red O stained areas in the images were quantified using ImageJ software (v1.52i).

**13. Western blot analysis**

AML12 cells were plated in 6-well plates at a density of 2×10^5^ cells per well and incubated overnight to allow for cell attachment. Then the cells were incubated with oleate (OA, 0.5 mM) and treated with free CEL, OPDEA-PCL/CEL micelles, or PEG-PCL/CEL micelles at CEL-equivalent concentration of 0.5 μg/mL for 48 h. Cell proteins were extracted via homogenization in ice-cold RIPA buffer supplemented with PMSF (Fdbio science, FD0100), protein and phosphatase inhibitor cocktail (Fdbio science, FD1001 and FD1002). The total protein concentration was measured using the BCA Protein Assay Kit (Millipore, USA). Subsequently, 20 μg aliquots of hepatic proteins were separated on 4~12% or 4~20% SDS-polyacrylamide gels (Genscript, China) through electrophoresis and then transferred to polyvinylidene fluoride (PVDF) membranes. Following blockage with 5% skim milk in TBST buffer, the membranes were subjected to overnight incubation with specific primary antibodies, succeeded by a 1-hour incubation with matching secondary antibodies. Detection of the blots was carried out using ECL detection reagents, with visualization performed on the FluorChem E System (ProteinSimple, USA), and analyzed by ImageJ software (v1.52i).

**14. Mucus penetration study**

A 500-μm-thick layer of rat intestinal mucus was applied to 12-well transwell polycarbonate inserts (membrane micropore size ≈ 0.4 μm). The micelles solution (100 μL, OPDEA-^Cy5^PCL/CEL micelles or PEG-^Cy5^PCL/CEL micelles, Cy5-eq. dose, 10 μg/mL) was introduced into the apical compartment, while 600 μL of PBS (pH 7.4) was added to the basolateral compartment. The transwell assembly was then incubated at 37 ℃ incubator. At specified time points, 100 μL solution was collected from the basolateral compartment and replaced with an equal volume of PBS. The concentration of the polymer in the sampled solution was measured using a microplate reader (SpectraMax iD5, Molecular Devices, USA) with excitation and emission wavelengths set at 640 nm and 660 nm, respectively.

**15. Transepithelial transport study *in vitro***

Caco-2 cells were seeded onto 12-well transwell polycarbonate cell culture inserts (membrane micropore size ≈ 0.4 μm, Corning, USA) with an approximate cell number of 5×10⁵ per well. Over the 2-week culture period, the culture media in both the apical and basolateral compartments were refreshed every other day. The integrity of the cell monolayer was assessed by measuring trans-endothelial electrical resistance (TEER) using an epithelial volt/ohm meter (Millicell ERS-2, Millipore). Once the TEER value surpassed 500 Ω·cm², the apical compartment's medium was replaced with 500 μL of fresh medium containing OPDEA-^Cy5^PCL/CEL micelles or PEG-^Cy5^PCL/CEL micelles (the Cy5-equivalent dose was 0.5 μg/mL), while the basolateral compartment received 1 mL of fresh medium. At set time points, 100 μL of the sample was taken from the basolateral compartment and replenished with the same volume of fresh medium. The Cy5-labelled micelle concentrations were determined by a microplate reader (SpectraMax iD5, Molecular Devices, USA) with excitation wavelengths at 640 nm and emission wavelengths at 660 nm, respectively.

**16. *In situ* absorption of Cy5-labeled micelles**

The intestinal absorption of OPDEA- ^Cy5^PCL/CEL or PEG- ^Cy5^PCL/CEL micelles were quantified by an intestinal loop method according to the reported procedures. SD rats were randomly assigned to two groups (n = 3) and were fasted for 12 h before the experiment, with free access to water. While anesthetized with pentobarbital, the rats received surgery to carefully expose the segments of jejunum with well-maintained mesenteric and capillary functions. A 10 cm region was tied off using surgical sutures, and OPDEA- ^Cy5^PCL/CEL or PEG- ^Cy5^PCL/CEL solution (5 mL, Cy5-eq. dose, 10 μg/ mL) were injected into the loop using a syringe. Following a 3-hour period, the rats were sacrificed and then the intestine segments were excised, rinsed with physiological saline and imaged with an IVIS Spectrum system. The total fluorescence intensity of the intestine segment was measured. The intestine segment was then fixated, dehydrated and frozen by embedding in O.C.T gel, and sectioned into slices of 8 μm thick using a Leica Microm CM3050S cryostat (Leica biosystems, Germany) and stained with DAPI for confocal microscopy imaging.

**17. The integrity of the OPDEA-PCL micelles evaluated by fluorescence resonance energy transfer (FRET) method**

**1) Synthesis of OPDEA-^Cy5^PCL and OPDEA-^Cy5.5^PCL.**

The polymer OPDEA-PCL-Boc underwent deprotection of the BOC group under trifluoroacetic acid (TFA) conditions, exposing the -NH₂ terminal group of PCL. Then a solution of Cy5-NHS or Cy5.5-NHS in DMSO (10 mg/mL) was added gradually into a solution of the OPDEA-PCL-NH_2_ in DMF (10 mg/mL) while stirring. The weight ratio of the fluorescent dye to the copolymer was kept at 1:100 (w/w). The solution was stirred for 12 h in the dark. To remove the unconjugated fluorescent dye, the solution was placed into a dialysis bag (MWCO 3500) and dialyzed against DMF. Then the solution was subsequently dialyzed against ddH₂O to remove DMF, followed by lyophilization to obtain the solid product OPDEA-^Cy5^PCL and OPDEA-^Cy5.5^PCL.

**2) Preparetion of OPDEA-PCL FRET micelles**

OPDEA-^Cy5^PCL and OPDEA-^Cy5.5^PCL were dissolved in DMF at 100 mg/mL and then added dropwise into ddH₂O under stirring to facilitate self-assembly into micelles. Then the solution was subsequently dialyzed against ddH₂O to remove DMF. The final micellar solution was adjusted to a concentration of 10 mg/mL. Emission spectra of FRET micelles excited at 640 nm upon dilution with ddH₂O, PBS, 10%FBS, SGF and SIF was measured by microplate reader (SpectraMax iD5, Molecular Devices, USA).

**3) The stability of the micelles in the intestinal epithelium cells.**

Caco-2 cells (1×10^5^ cells/well) were seeded in glass-bottom culture dishes and incubated overnight with OPDEA-PCL FRET micelles (Cy5/Cy5.5-eq. dose, 0.5 μg/mL). After 2, 4 and 6 h, the nuclei were stained with Hoechst 33342 (2 μM, Invitrogen) for 15 min. The cells were then washed three times with PBS and imaged using an Olympus FV3000 confocal microscope (Olympus, Japan), and emission spectra was collected by Lambda mode. Fluorescence emission spectra was collected from five randomly selected sites of interest (ROIs) within the cells.

**4) The stability of the micelles in the intestin.**

The intestinal absorption of FRET micelles were quantified by an intestinal loop method as mentioned before. OPDEA-PCL FRET micelles (Cy5/Cy5.5-eq. dose, 10 μg/mL) were injected into the intestinal loop. After 3 or 12 h, the rats were sacrificed and then the intestine segments were collected. The intestine segments were then fixated, dehydrated and frozen by embedding in O.C.T gel, Subsequently, they were sectioned into slices and stained with DAPI for confocal microscopy imaging. Emission spectra was collected by Lambda mode and from five randomly selected sites of interest (ROIs) within the cells.

**5) The integrity of the OPDEA-PCL micelles in the blood circulation.**

C57BL/6J mice (6-8 weeks old) were randomly divided into two groups (n = 3) and were fasted for 12 h before the experiment, with free access to water. OPDEA-PCL FRET micelles were delivered to mice by oral gavage (Cy5/Cy5.5-eq. dose, 10 μg/mL, 200μL). At 3, 6, 12, and 24 h post-administration, the serum was harvested. The emission spectra of serum excited at 640 nm was measured by microplate reader (SpectraMax iD5, Molecular Devices, USA).

**18. *In vivo* biodistribution**

C57BL/6J mice (6-8 weeks old) were randomly divided into two groups (n = 3) and were fasted for 12 h before the experiment, with free access to water. DiR-loaded free CEL, OPDEA-PCL/CEL or PEG-PCL/CEL micelles were delivered to mice by oral gavage at a 100 μg/kg DiR-eq. dose. At 3, 6, 12, and 24 h post-injection, the mice were anesthetized and then imaged under an IVIS Spectrum system. The mice were subsequently euthanized, and the whole gastrointestinal tract along with major organs (heart, liver, spleen, lungs, kidneys) were harvested for *in vivo* fluorescence imaging. The fluorescence intensity in each gastrointestinal tract and organ was measured using the IVIS Spectrum Software. C57BL/6J mice fed with LFD or HFD (n = 3) were orally administered with DiR-loaded OPDEA-PCL/CEL micelles at a 100 μg/kg DiR-eq. dose. At 6 h after the injection, the mice were anesthetized and then imaged as mentioned above.

**19. Preparation of biological sample for LC-MS analysis**

The liver suspension was obtained by homogenization with a tissue homogenizer (Shanghai Jingxin Experimental Technology, China) with ddH_2_O at a ratio of 1:2 (w/w). The plasma or organ suspension (100 μL) was lyophilized. The residue was extracted using 100 μL methanol, mixed thoroughly by vortexing for 1 min, and then centrifuged at 15,000 rpm for 15 min. The supernatant was collected for LC-MS analysis.

**20. Pharmacokinetics of free CEL and micelles**

Twelve SD rats were fasted 12 h before the experiment with free access to water and randomly separated into three groups (n = 4). Free CEL, OPDEA-PCL/CEL micelles or PEG-PCL/CEL micelles were given to rats by oral gavage at a 1 mg/kg CEL-equivalent dose. At timed intervals, blood samples (0.3 mL) were collected from the orbital venous plexus and centrifuged at 2000×g for 10 min at 4 ℃ to obtain the plasma. The plasma was mixed with methanol at a ratio of 1:4 (v/v) to precipitate the proteins. The mixture was centrifuged at 12,000×g for 20 min and the supernatant was collected. The CEL concentration in the supernatant was measured by LC-MS as mentioned above.

**21. Micelles uptake in hepatic subcellular populations**

The MASLD mouse model was established as previously described. These mice were orally administered with 200 μL of OPDEA-^Cy5^PCL/CEL micelles per mouse (Cy5-eq. 10 μg/mL). After 12 h, the livers were harvested for analysis. The liver tissue was finely chopped and digested in a 0.1% w/v collagenase IV solution at 37 ℃, with gentle shaking at 90 rpm for 30 min. After digestion, the cell suspension was gently triturated and filtered through a 70 μm cell strainer to yield a single-cell suspension. Cell types were distinguished by flow cytometry based on size (FSC), granularity (SSC), and specific surface markers as follows: hepatocytes (high FSC and SSC, CD45^-^, CD31^-^), endothelial cells (CD45^-^, CD31^+^), leukocytes (CD45^+^), and Kupffer cells (CD45^+^, F4/80^high^, CD11b^low^). The percentage of Cy5^+^ cells within each cell population was measured and quantified. Flow cytometry was used to analyze the internalization of OPDEA-^Cy5^PCL/CEL micelles by different hepatic subcellular populations.

Immunofluorescence staining was performed on frozen liver sections. Liver tissues were immersed in optimal cutting temperature (O.C.T.) compound and immediately subjected to flash-freezing. Cryosections were cut into 8 μm slices using a Leica CM1950 cryostat (Leica Microsystems, Germany) and mounted onto glass slides. The sections underwent fixation in 4% PFA for 10 min at room temperature, and subsequently underwent three rinses with PBS. Permeabilization was performed using 0.1% Triton X-100 in PBS for 10 min, followed by blocking with 5% goat serum in PBS for 1 h at RT. Primary antibodies, including F4/80 (1:200 in PBS containing 1% goat serum, Kupffer cell marker, membrane protein) and HNF4α (1:200 in PBS containing 1% goat serum, hepatocyte marker, nuclear protein), were incubated overnight at 4 °C. After incubation, the sections were rinsed three times with PBS. They were then incubated with Goat Anti-Rabbit IgG H&L (Alexa Fluor® 488) (Abcam) at a 1:500 dilution in PBS supplemented with 1% goat serum for 1 h at RT in the dark. Following PBS washes, the sections were mounted with ProLong™ Gold Antifade Mountant with DAPI (ThermoFisher). Imaging was performed using an Olympus FV3000 confocal microscope (Olympus, Japan).

**22. Interaction between OPDEA-PCL/CEL micelles and HDL**

**1) OPDEA-PCL/CEL micelle interaction with HDL**

OPDEA-PCL/CEL micelles were incubated with varying concentrations of HDL while maintaining the micelles concentration constant at 200 μg/mL of OPDEA-PCL in pH 7.4 PBS. The HDL solution was serially diluted to achieve final concentrations at 0, 40, 80, 120, 160, 200, 250, 333, 500 and 1000 μg/mL. The micelles were then mixed with the HDL solution at an equal volume and incubated at 37 ℃ for 1 h with gentle shaking. The size distribution and zeta potential of micelles in the mixture were assessed using a Zetasizer Nano ZS90 (Malvern Instruments, UK).

**2) Fluorescence quenching assay to assess the interaction between HDL and OPDEA-PCL/CEL micelles**

100 μL of HDL solution (200 μg/mL) was mixed with OPDEA-PCL/CEL micelles at varied concentrations (0, 40, 80, 120, 160, 200, 250, 333, 500 and 1000 μg/mL, OPDEA-PCL eq.) in PBS. The mixtures were maintained at 37℃ for 1 h with gentle shaking. The inherent fluorescence excited at 285 nm of the HDL protein was measured using a microplate reader (SpectraMax iD5, Molecular Devices, USA) (excitation wavelength: 285 nm; emission wavelength range: 300-500 nm) in a black opaque microplate. Fluorescence quenching ratio was quantified by comparing the fluorescence intensities of HDL in the presence/abscence of different concentrations of OPDEA-PCL/CEL micelles. The quenching data were analyzed using the Stern-Volmer equation to determine the quenching constant.

**3) Native polyacrylamide gel electrophoresis (Native-PAGE) assay to assess the interaction between HDL and OPDEA-PCL/CEL micelles**

The HDL solution was prepared to 200 μg/mL in PBS, and OPDEA-PCL/CEL micelles were added at varied concentrations at 0, 40, 80, 120, 160, 200, 250, 333, 500 and 1000 μg/mL (OPDEA-PCL eq.). The mixtures were maintained at 37 °C for 1 h with gentle shaking. The samples were mixed with non-denaturing sample buffer. The samples were then loaded onto a 4-20% gradient polyacrylamide gel and subjected to electrophoresis at 4 °C. The electrophoresis was carried out using a Tris-glycine running buffer without SDS to maintain native conditions. After electrophoresis, the gels were stained using Coomassie Brilliant Blue to visualize the protein bands. The migration patterns of HDL in the presence of increasing concentrations of OPDEA-PCL/CEL micelles were analyzed to assess the HDL mobility.

**4) ITC experiments**

ITC was conducted using a NANO ITC calorimeter (TA Instruments, USA) at 37 °C under atmospheric pressure, with 25 sequential injections performed for each experiment. Each injection had a volume of 2 µL, and the OPDEA-PCL/CEL micelles (with an OPDEA-PCL equivalent concentration of 5 mg/mL) were injected into a cell containing different titration targets: HDL (0.5 mg/mL), BSA (0.5 mg/mL), phosphatidylcholine (0.5 mg/mL), or Apo-A1 (0.5 mg/mL). Each titration produced a heat profile. The effective volume of the lower chamber was 170 µL. To calculate the net reaction heat, the dilution heat was subtracted from the heat measured in the titration experiments. The resulting data were then analyzed using NanoAnalyze software (TA Instruments, USA).

**23. Evaluation of therapeutic efficacy of CEL-micelles in mouse MASLD model**

The MASLD mice were randomly assigned to four groups: 1) HFD control group, 2) free CEL group, 3) PEG-PCL/CEL micelles group, 4) OPDEA-PCL/CEL micelles group. The group of normal mice fed with LFD was served as the control group. Each group received oral administration every two days, with CEL-equivalent dose of 2 mg/kg in 200 μL volume per mouse. The HFD and LFD control groups were given 200 μL of ddH_2_O every two days. During the treatment period, body weight and food intake of each mouse were tracked to assess general health and impact of the treatments. Before the study concluded, hepatic steatosis was assessed via ultrasound imaging, with the liver-to-kidney contrast ratio employed as a quantitative indicator of fatty liver severity. Upon completion of the experiment, the mice were euthanized, and their body images were captured. Blood samples were drawn for serum biochemical analysis, and major organs and tissues, including heart, lungs, liver, spleen, kidneys, abdominal fat, epididymis and the colon, were harvested and analyzed. The liver and abdominal fat were photographed, and samples were preserved for further histological and biochemical analyses.

**24. Safety evaluation of CEL-micelles after oral treatment**

The samples from the aforementioned MASLD treatment animal experiment were collected and used. For the purpose of conducting complete blood counts and biochemical analysis, whole blood and serum were obtained from the mice. Biochemical indices, including alanine aminotransferase (ALT), aspartate aminotransferase (AST), gamma-glutamyl transferase (γ-GT), total cholesterol (TC), triglycerides (TG), total bilirubin (TBIL), direct bilirubin (DBIL), high-density lipoprotein cholesterol (HDL-C), low-density lipoprotein cholesterol (LDL-C), glucose (GLU), creatinine (CR), uric acid (UA), creatine kinase (CK), blood urea nitrogen (BUN), lactate dehydrogenase (LDH) and creatine kinase-MB (CK-MB), were detected to assess liver function, kidney function, and heart function respectively. Meanwhile, major organs, including heart, lungs, liver, spleen, kidneys, epididymis, and colon, were excised, fixed with 4% PFA, and sectioned for H&E staining.

To further evaluate safety, normal C57 mice were divided into four groups and were orally administered with ddH2O, free CEL, OPDEA-PCL/CEL micelles or PEG-PCL/CEL micelles, respectively, at a dose of 2 mg/kg CEL-eq. The treatment was administered every other day for a total of 3 weeks. For the purpose of conducting complete blood counts and biochemical analysis, whole blood and serum were obtained from the mice.

**Reference**

[1] a)T. Fang, H. Wang, X. Pan, P. J. Little, S. Xu, J. Weng, *Int J Biol Sci* **2022**, 18, 5681; b)P. Xie, Y. Peng, L. Qiu, *Carbohydr Polym* **2022**, 288, 119388.

[2] W. Fan, Q. Wei, J. Xiang, Y. Tang, Q. Zhou, Y. Geng, Y. Liu, R. Sun, L. Xu, G. Wang, Y. Piao, S. Shao, Z. Zhou, J. Tang, T. Xie, Z. Li, Y. Shen, *Adv Mater* **2022**, 34, e2109189.


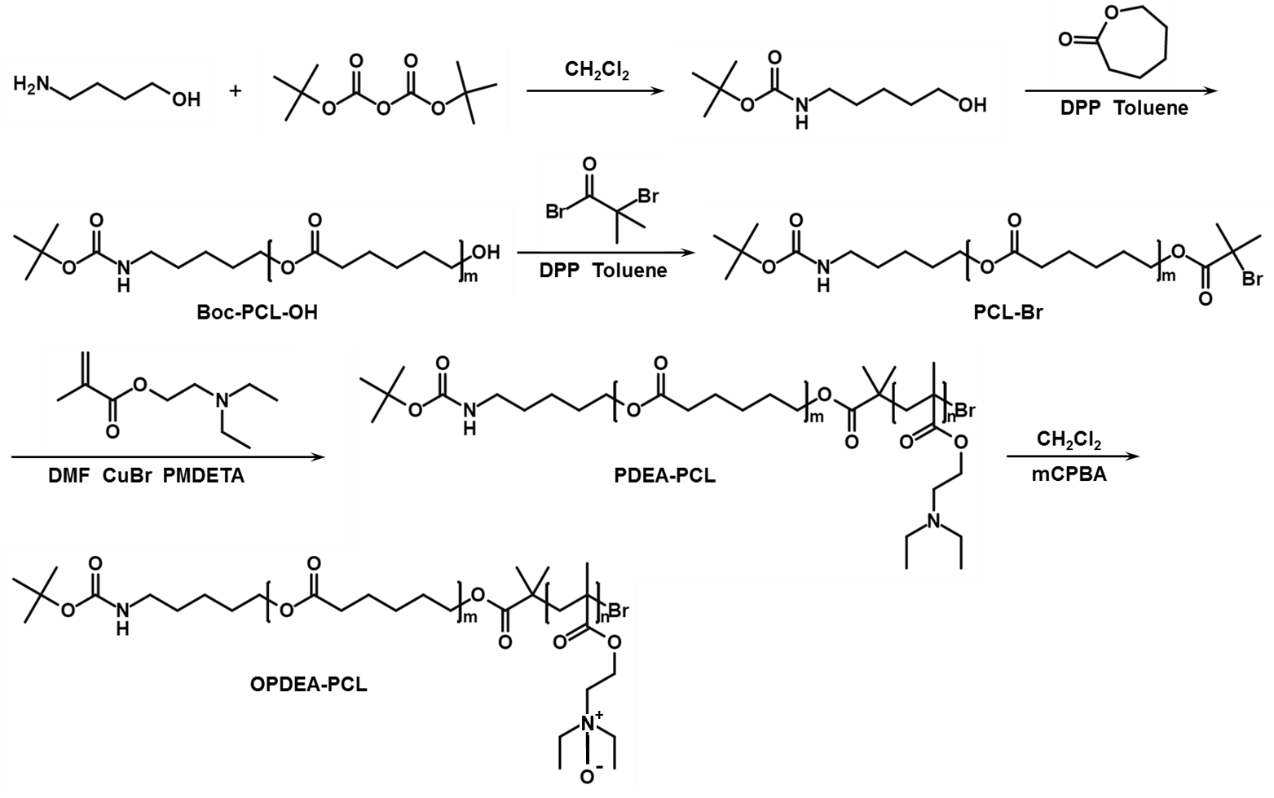


**Figure S1**. Synthesis route of OPDEA-PCL.


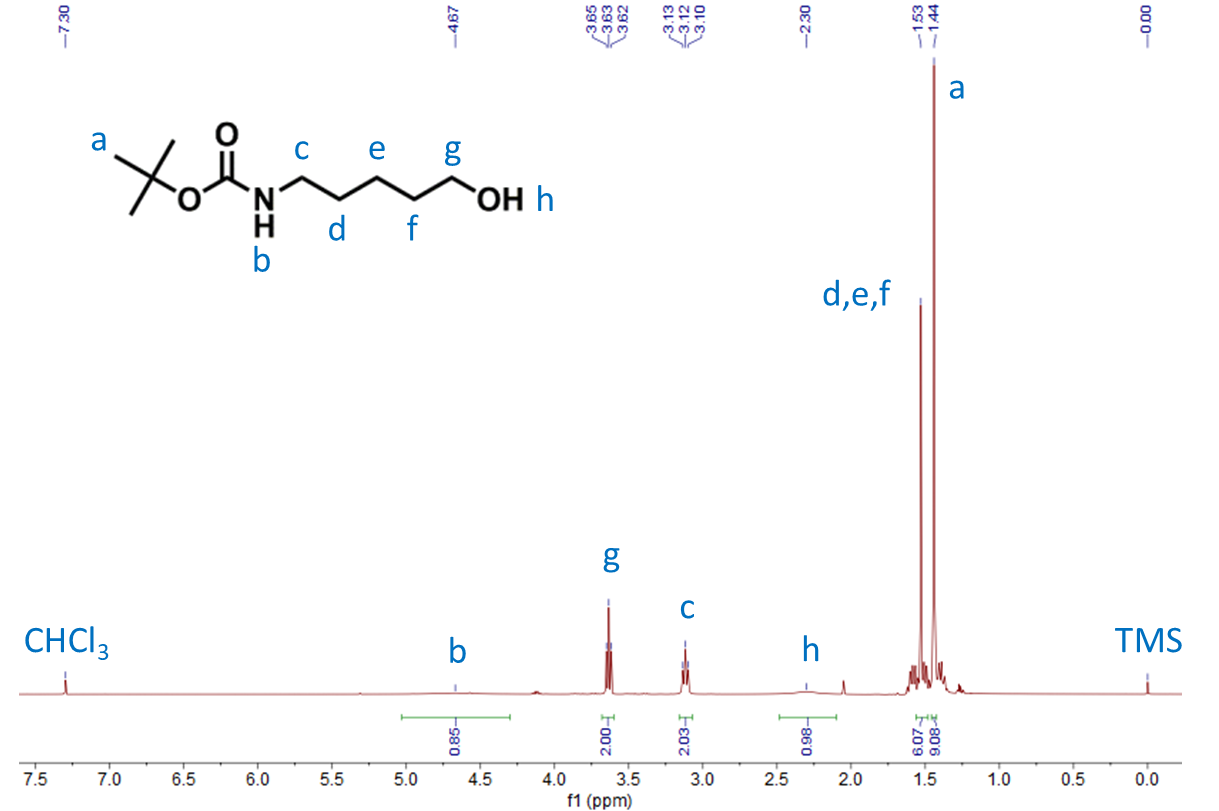


**Figure S2**. The ^1^H-NMR spectrum of 5-(Boc-amino)-1-pentanol in CDCl_3_.


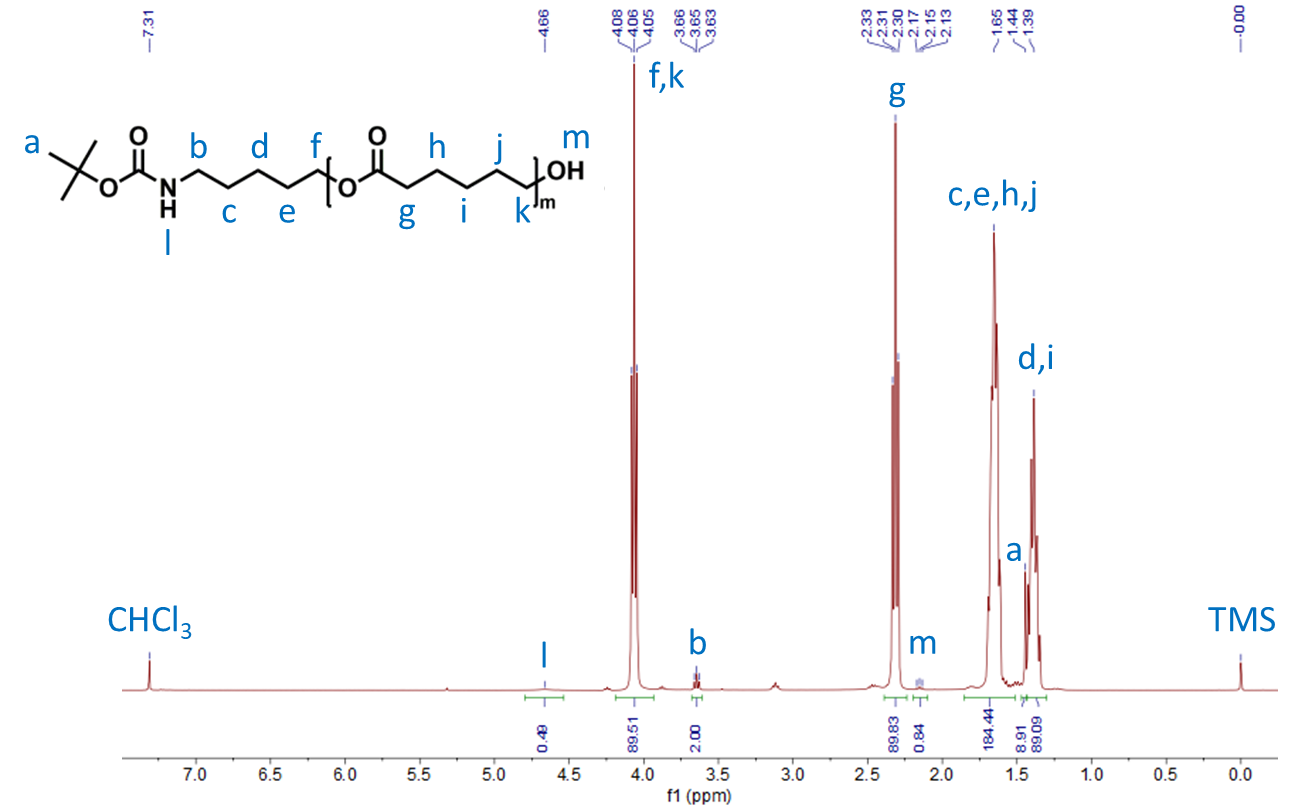


**Figure S3**. The ^1^H-NMR spectrum of BocNH-PCL-OH in CDCl_3_.


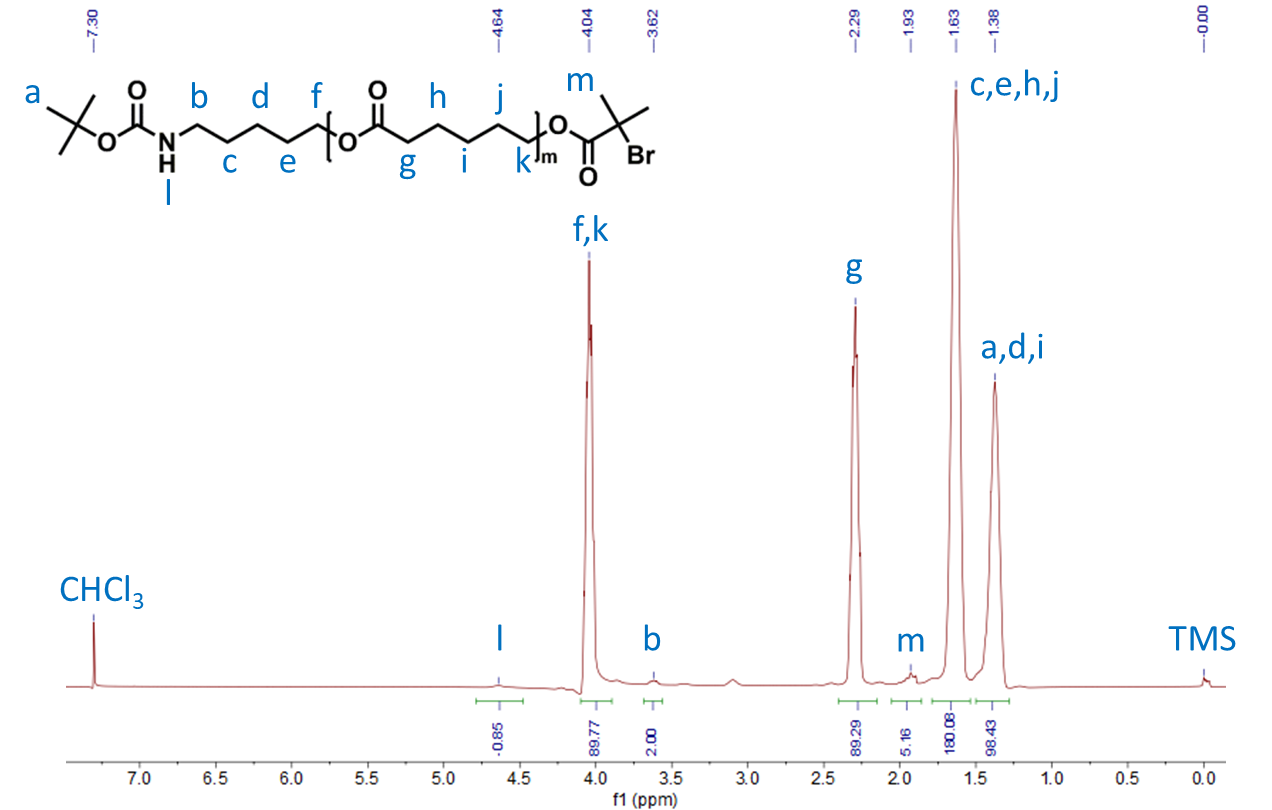


**Figure S4**. The ^1^H-NMR spectrum of PCL-Br in CDCl_3_.


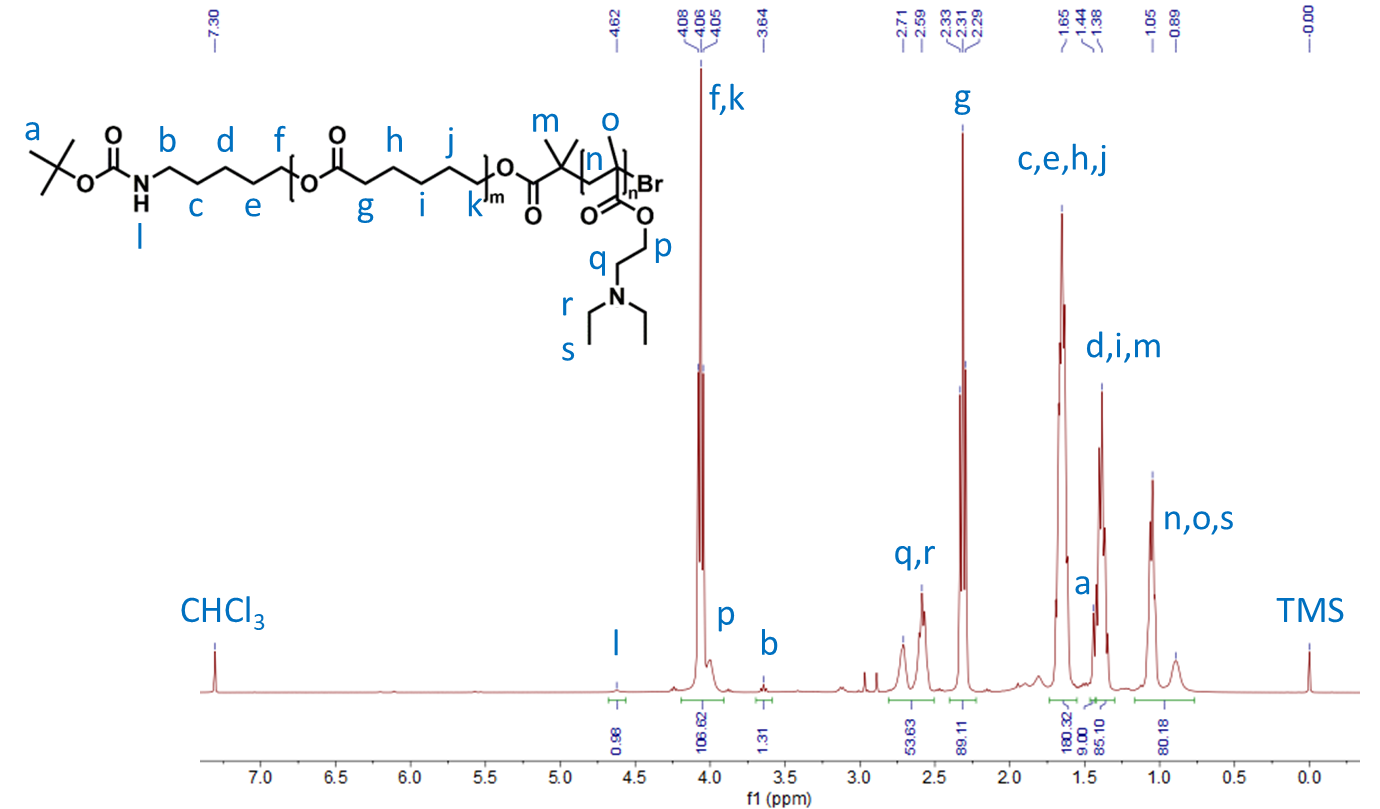


**Figure S5**. The ^1^H-NMR spectrum of PDEA-PCL in CDCl_3_.


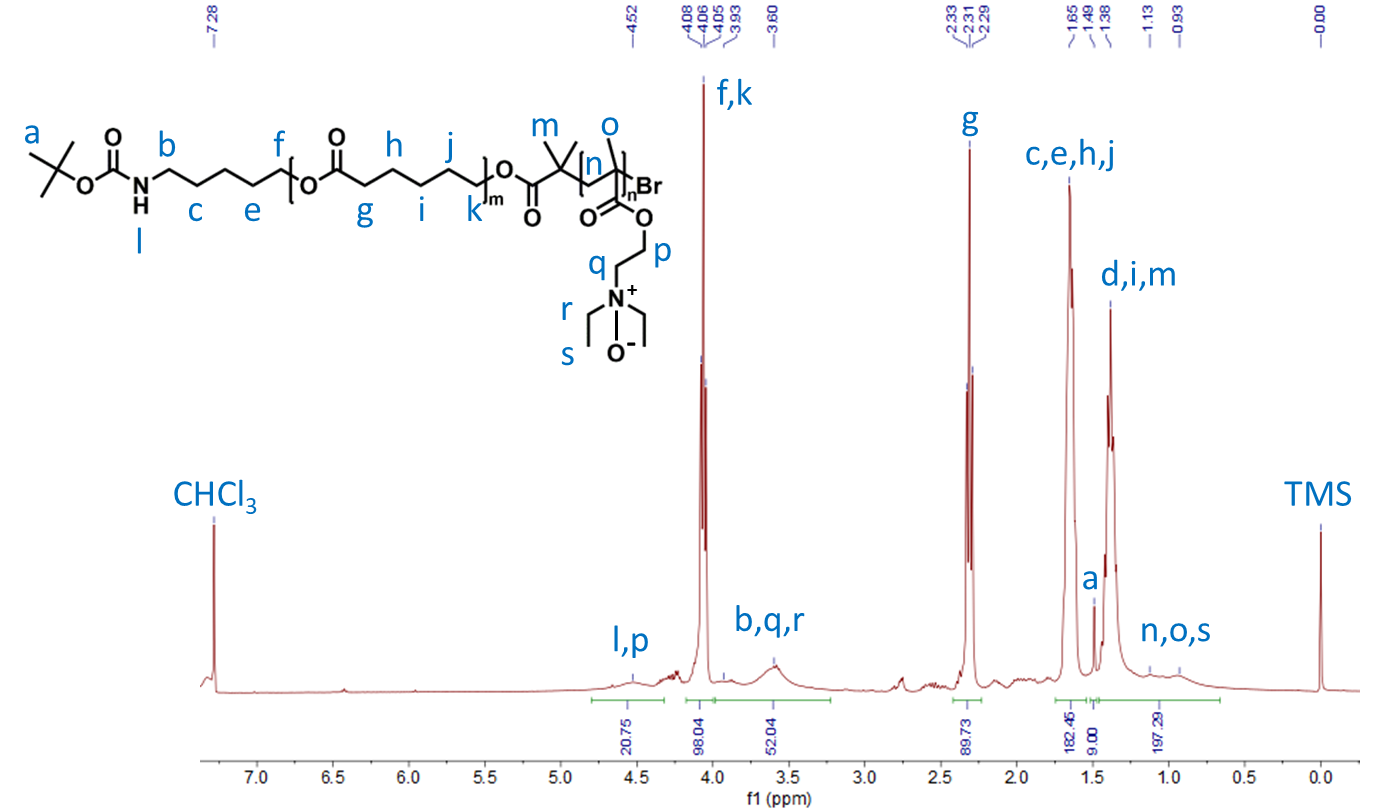


**Figure S6**. The ^1^H-NMR spectrum of OPDEA-PCL in CDCl_3_.


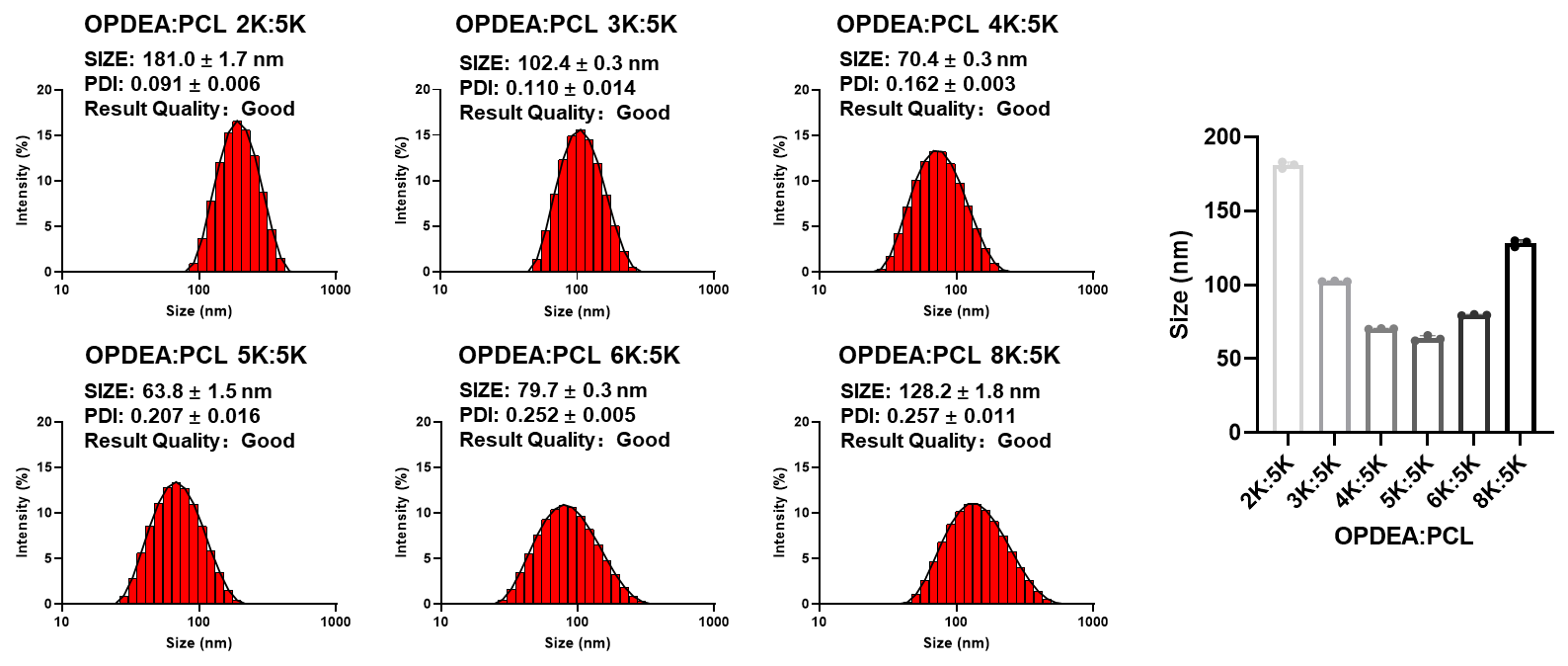


**Figure S7**. The sizes of OPDEA-PCL/CEL micelles at different block ratios of OPDEA to PCL characterized by Dynamic light scattering (DLS) (n = 3).


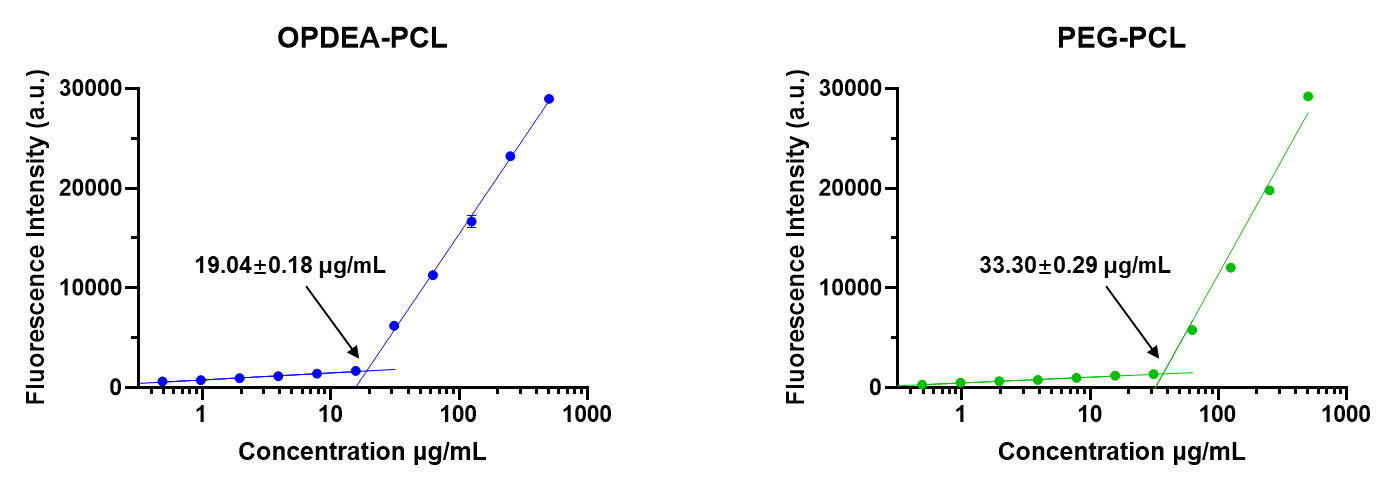


**Figure S8**. Critical micelle concentrations of OPDEA-PCL micelles and PEG-PCL micelles determined by the Nile red method (n = 3).


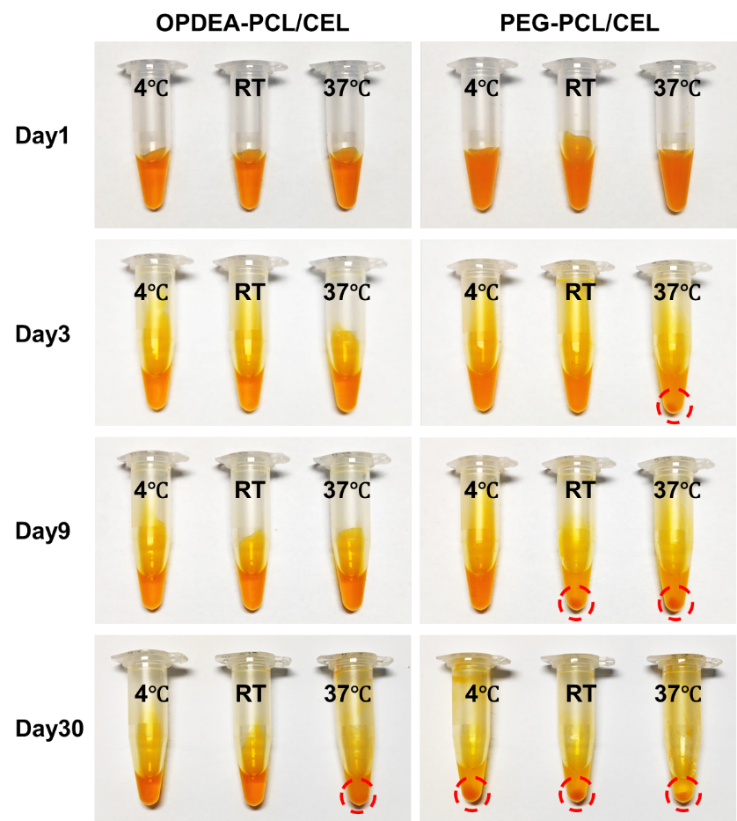


**Figure S9.** Stability of oral formulation of the OPDEA-PCL/CEL micelles and PEG-PCL/CEL micelles solutions after timed storage at 4°C, room temperature, and 37°C for long-term stability tests.


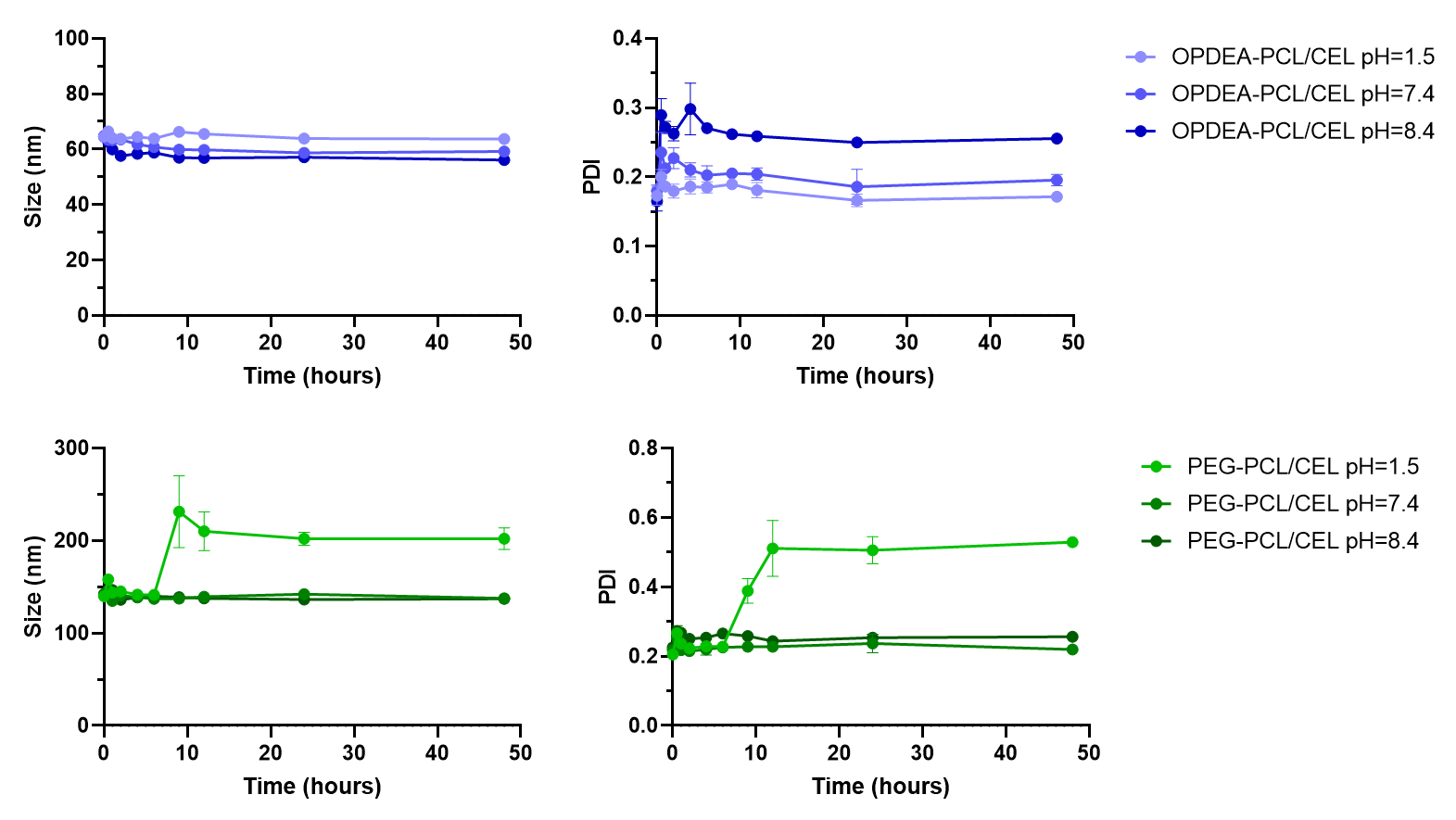


**Figure S10**. The sizes of OPDEA-PCL/CEL micelles and PEG-PCL/CEL micelles in the simulated physiological media (simulated gastric fluid, pH = 1.5; simulated intestinal fluid, pH = 8.4) and PBS (pH = 7.4) characterized by DLS (n = 3).


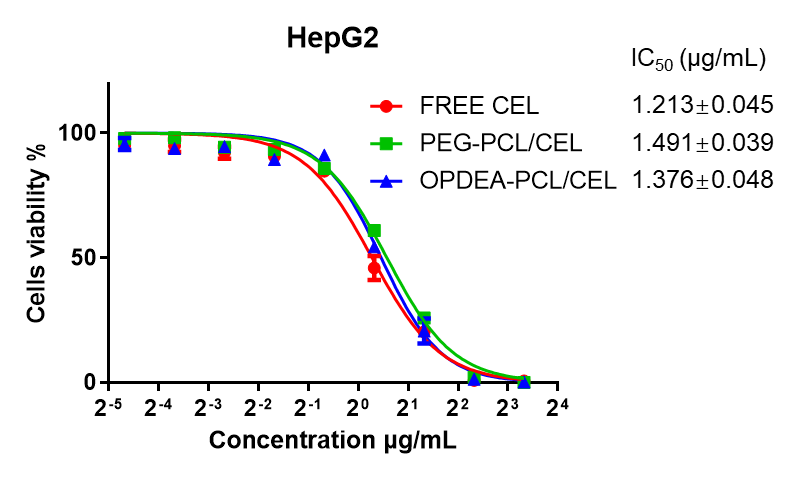


**Figure S11**. Cytotoxicity of free CEL, OPDEA-PCL/CEL micelles and PEG-PCL/CEL micelles on HepG2 cells after incubation for 24 h (n = 3).


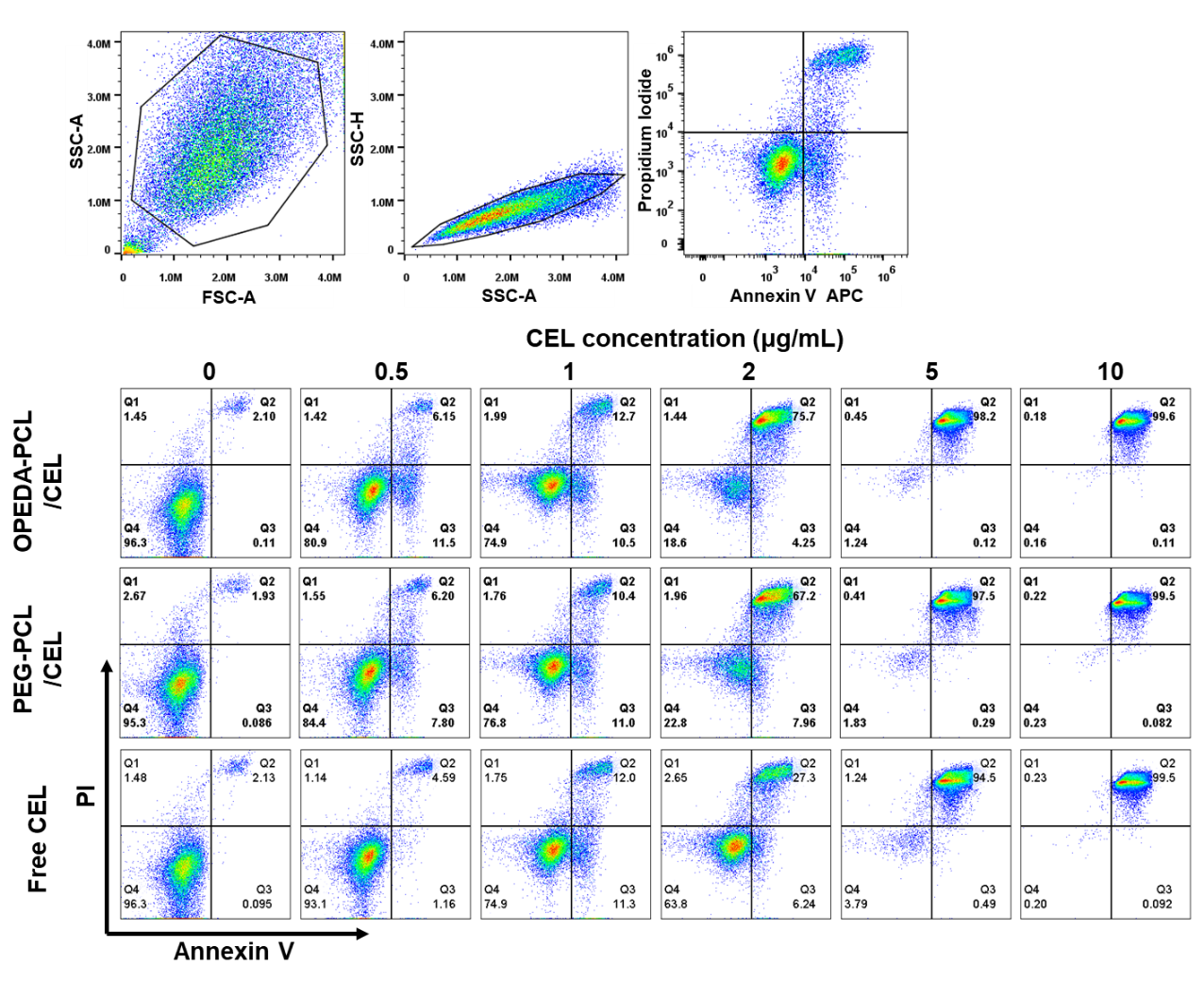


**Figure S12**. Flow cytometry gating strategy for apoptotic cells (Annexin V^+^ PI^+^ cells) of AML12 cells after incubation with free CEL, OPDEA-PCL/CEL micelles and PEG-PCL/CEL micelles for 24 h.


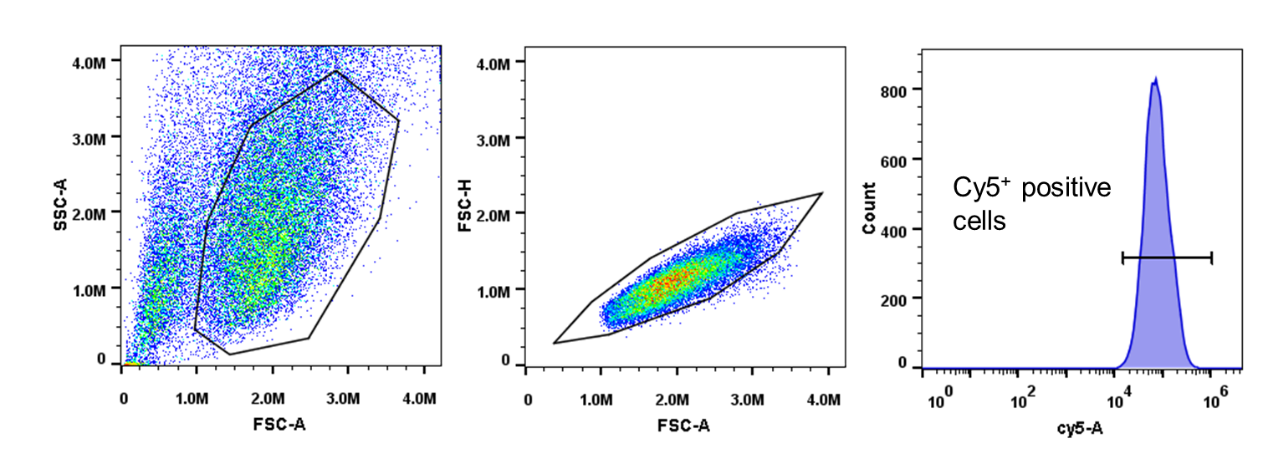


**Figure S13**. Flow cytometry gating strategy for cellular uptake rates of OPDEA-^Cy5^PCL/CEL micelles and PEG-^Cy5^PCL/CEL micelles (Cy5-eq. dose of 0.5 µg/mL) on AML12 cells at different time points (0.5 h, 1 h, 2 h, 4 h, 6 h), see Fig. 2A.


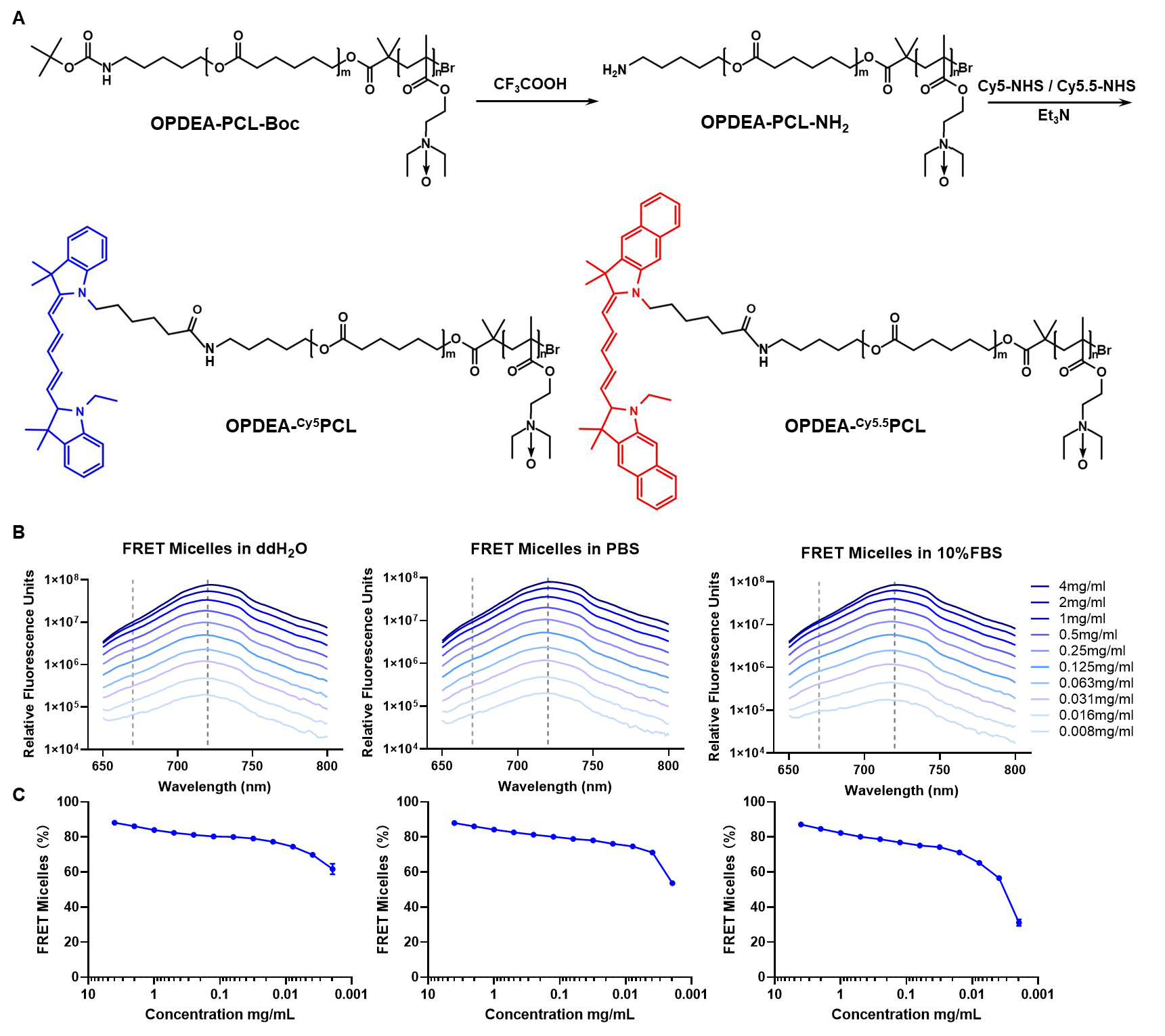


**Figure S14.** The stability assessment of the OPDEA-PCL using FRET micelles. (A) Synthesis route of OPDEA-^Cy5^PCL and OPDEA-^Cy5.5^PCL. (B) Emission spectra of FRET micelles excited at 640 nm upon dilution with ddH₂O, PBS and 10%FBS. (C) The micelle integrity is estimated by the FRET efficiency. The FRET efficiency was calculated as Intensity_Cy5.5_ / (Intensity_Cy5.5_ + Intensity_Cy5_).


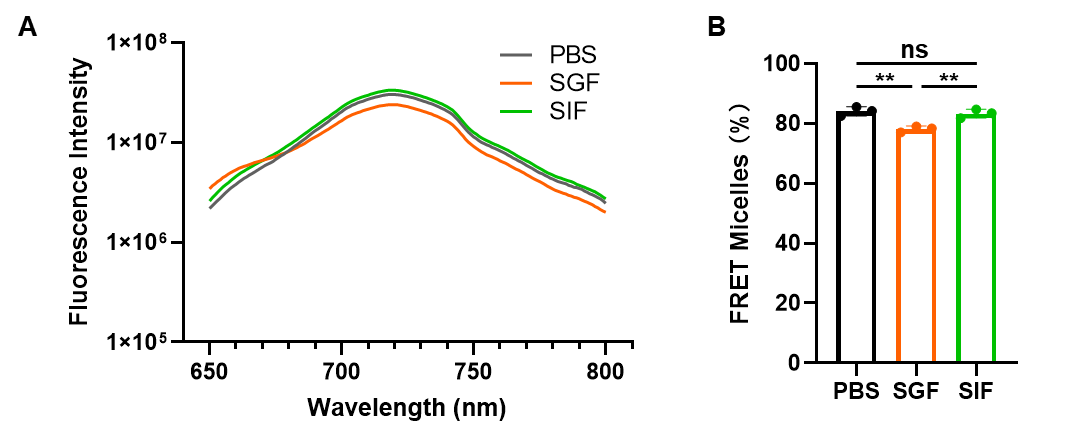


**Figure S15.** The stability of OPDEA-PCL FRET micelles in simulated physiological media. (**A**) Fluorescence emission spectra of FRET micelles (Cy5/Cy5.5 eq.10 μg/mL) excited at 640 nm in PBS, SGF and SIF. (**B**) The micellar integrity estimated by the FRET efficiencies (presented as mean ± SD, n=3).


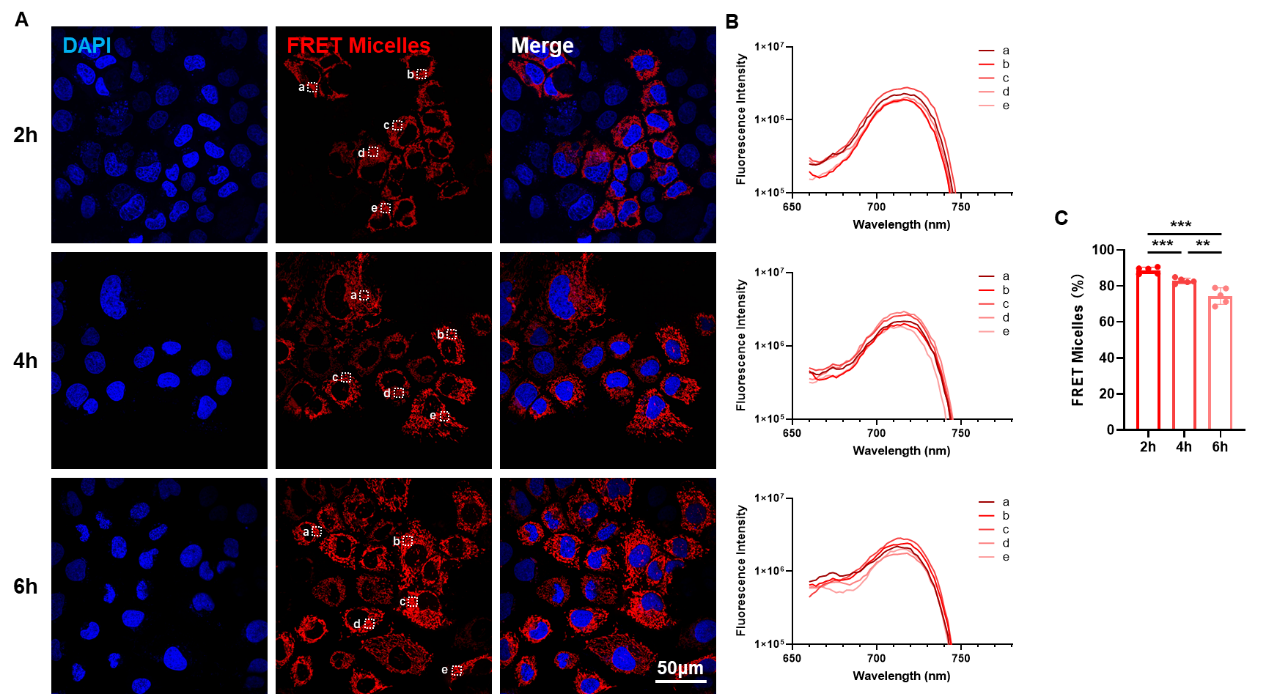


**Figure S16.** Intracellular stability of OPDEA-PCL FRET micelles in Caco-2 cells. (A) Confocal microscopy images of Caco-2 cells incubated with FRET micelles (Cy5/Cy5.5 eq. 0.5 μg/mL) for 2, 4 and 6 h. (B) Fluorescence emission spectra collected from five randomly selected sites of interest (ROIs) within the cells. (C) The micellar integrity estimated by the FRET efficiencies (presented as mean ± SD, n=5).


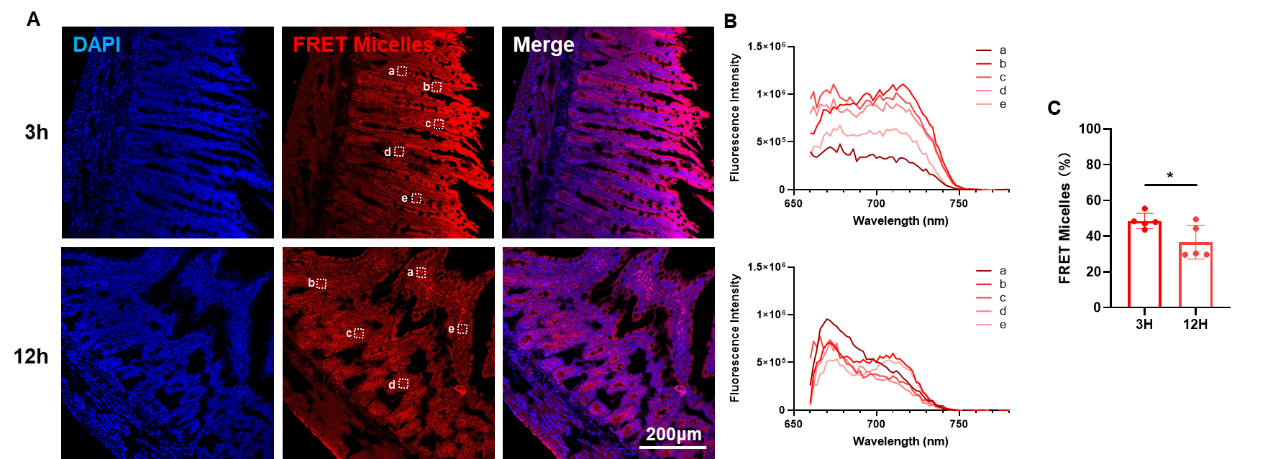


**Figure S17**. Observation of OPDEA-PCL FRET micelles *in vivo*. (A) Confocal microscopy images of intestinal cross-sections perfused with OPDEA-PCL FRET micelles (Cy5/Cy5.5 eq. 10 μg/mL) for 3 and 12 h. (B) Fluorescence emission spectra collected from five randomly selected regions of interest (ROIs). (C) Quantitative analyses of micellar integrity. The corresponding FRET efficiencies were calculated as Intensity_Cy5.5_ / (Intensity_Cy5.5_ + Intensity_Cy5_). (presented as mean ± SD, n=5).


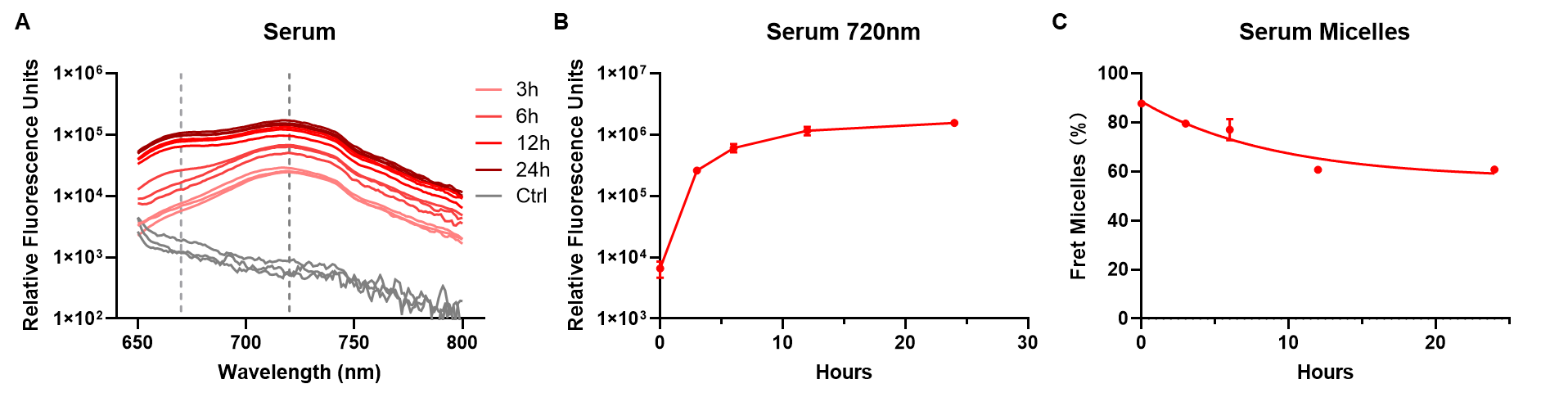


**Figure S18**. *In vivo* tracking of OPDEA-PCL FRET micelles in serum after oral administration. (A) The fluorescence emission spectra of the serum at timed intervals after the oral administration. The mice were orally administered with 200 μL of OPDEA-PCL FRET micelles (Cy5/Cy5.5 eq. 10 μg/mL), and the mouse serum was collected at 3, 6, 12, and 24 h post-administration. The emission spectra were recorded under 640 nm excitation. (B) Time-dependent FRET signal intensity. (C) The intact micelle percentage by the FRET efficiency calculated as Intensity_Cy5.5_ / (Intensity_Cy5.5_ + Intensity_Cy5_) (presented as mean ± SD, n = 3).


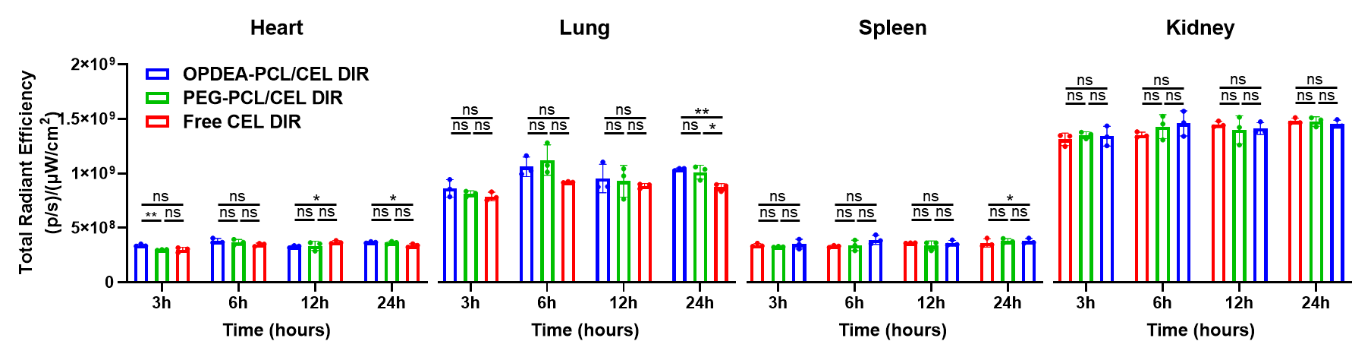


**Figure S19**. *Ex vivo* living imaging quantification of fluorescence intensity of hearts, lungs, spleens and kidneys at different time points post administration (n = 3).


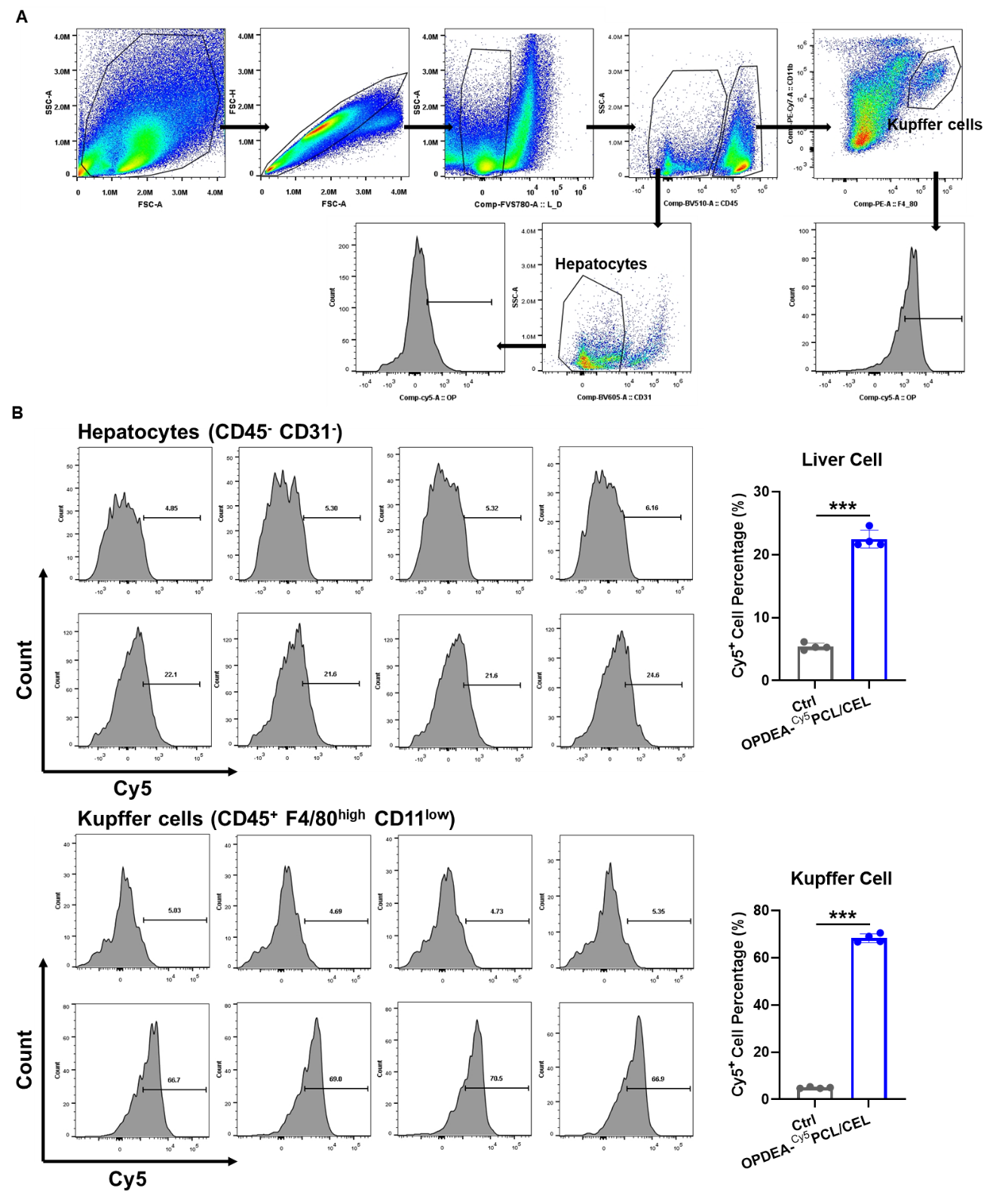


**Figure S20**. (**A**) Flow cytometry gating strategy for hepatocyte (CD45^-^, CD31^-^) and Kupffer cells (CD45^+^, F4/80^high^, CD11b^low^) identification. (**B**) The uptake of OPDEA-^Cy5^PCL/CEL micelles (Cy5-eq. dose of 10 µg/mL) in hepatic subcellular populations measured by flow cytometry, and the corresponding quantitation of Cy5 positive cells (n = 4).


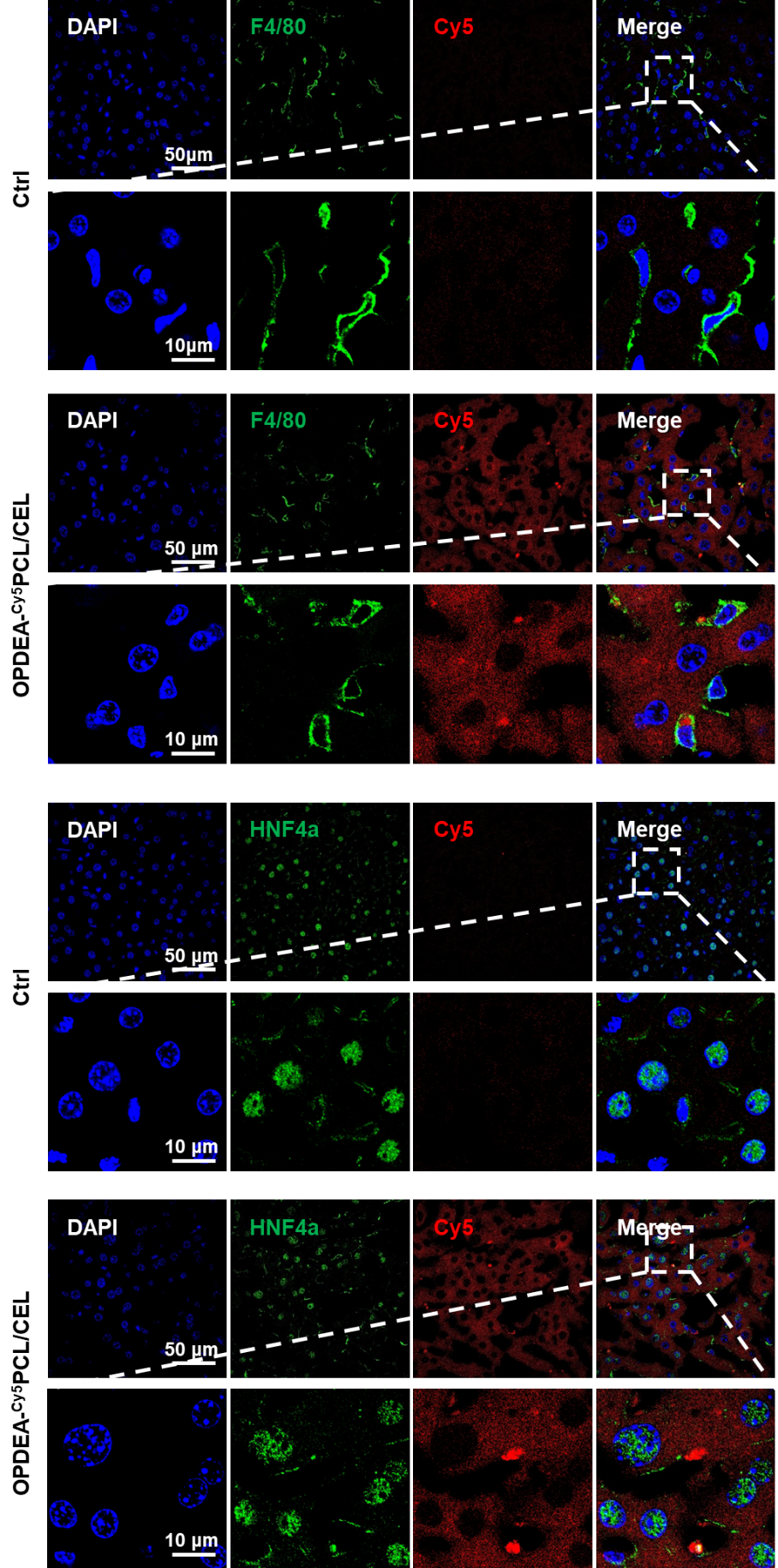


**Figure S21.** Cellular uptake of OPDEA-^Cy5^PCL/CEL micelles (Cy5-eq. dose of 10 µg/mL) in hepatocytes (HNF4a^+^) and Kupffer cells (F4/80^+^) imaged by a laser scanning confocal microscope.


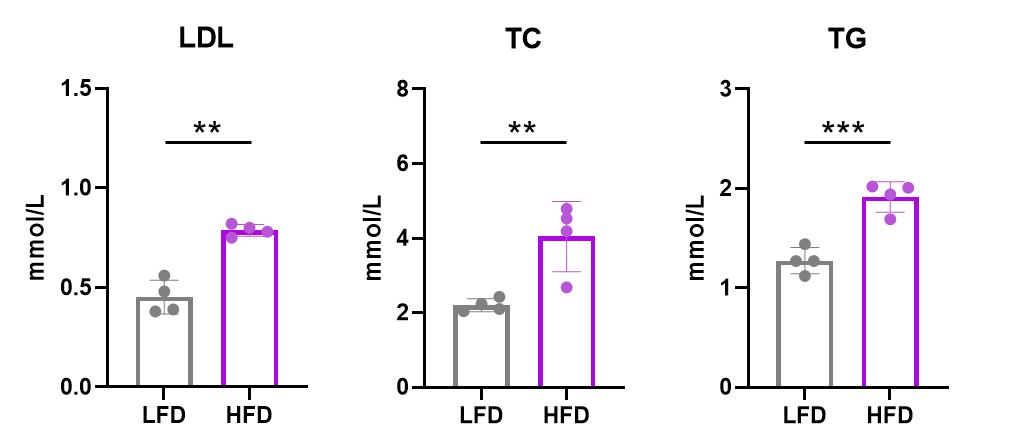


**Figure S22**. The serum biochemical analysis of LDL, TC and TG contents in plasma of C57 mice fed with LFD or HFD (n = 4).


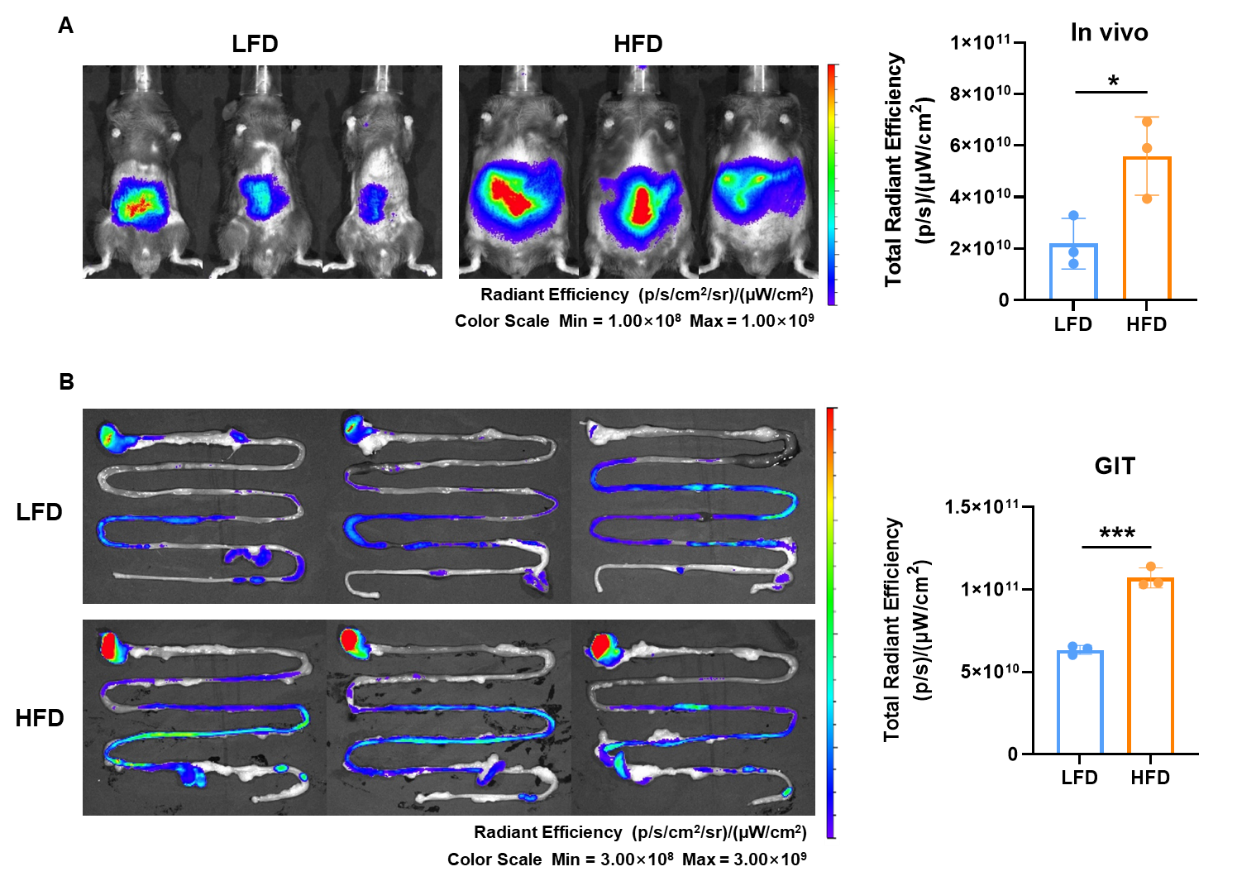


**Figure S23.** Living imaging of the LFD or HFD mice (A) and *ex vivo* imaging of their GIT (B) at 12 h post oral administration of DiR-loaded OPDEA-PCL/CEL micelles (DiR-eq. dose, 0.1 mg/kg) and quantification of the fluorescence intensity (n = 3).


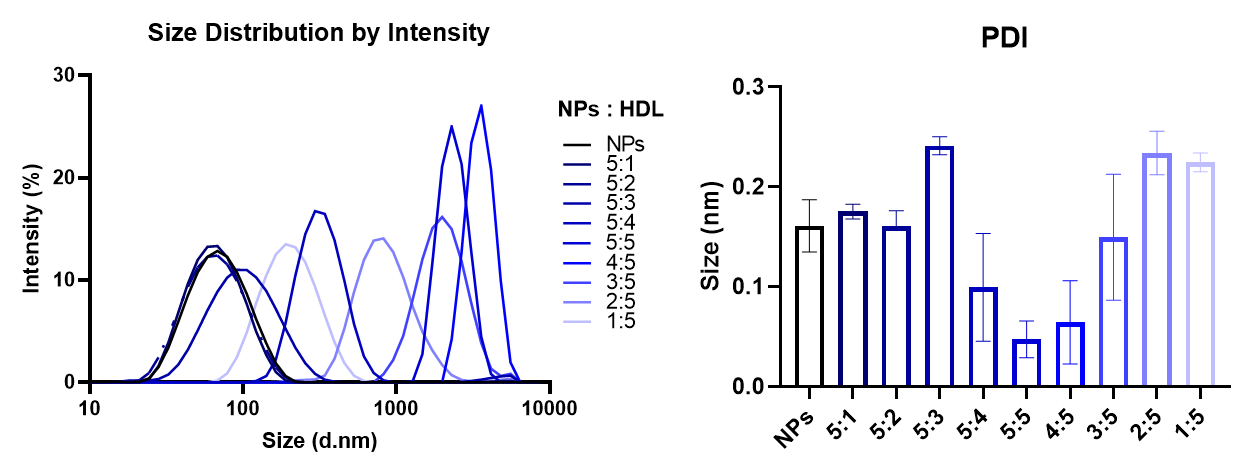


**Figure S24**. The size distribution and PDI of OPDEA-PCL/CEL micelles after incubation with HDL protein at different mass ratios measured by DLS (n = 3).


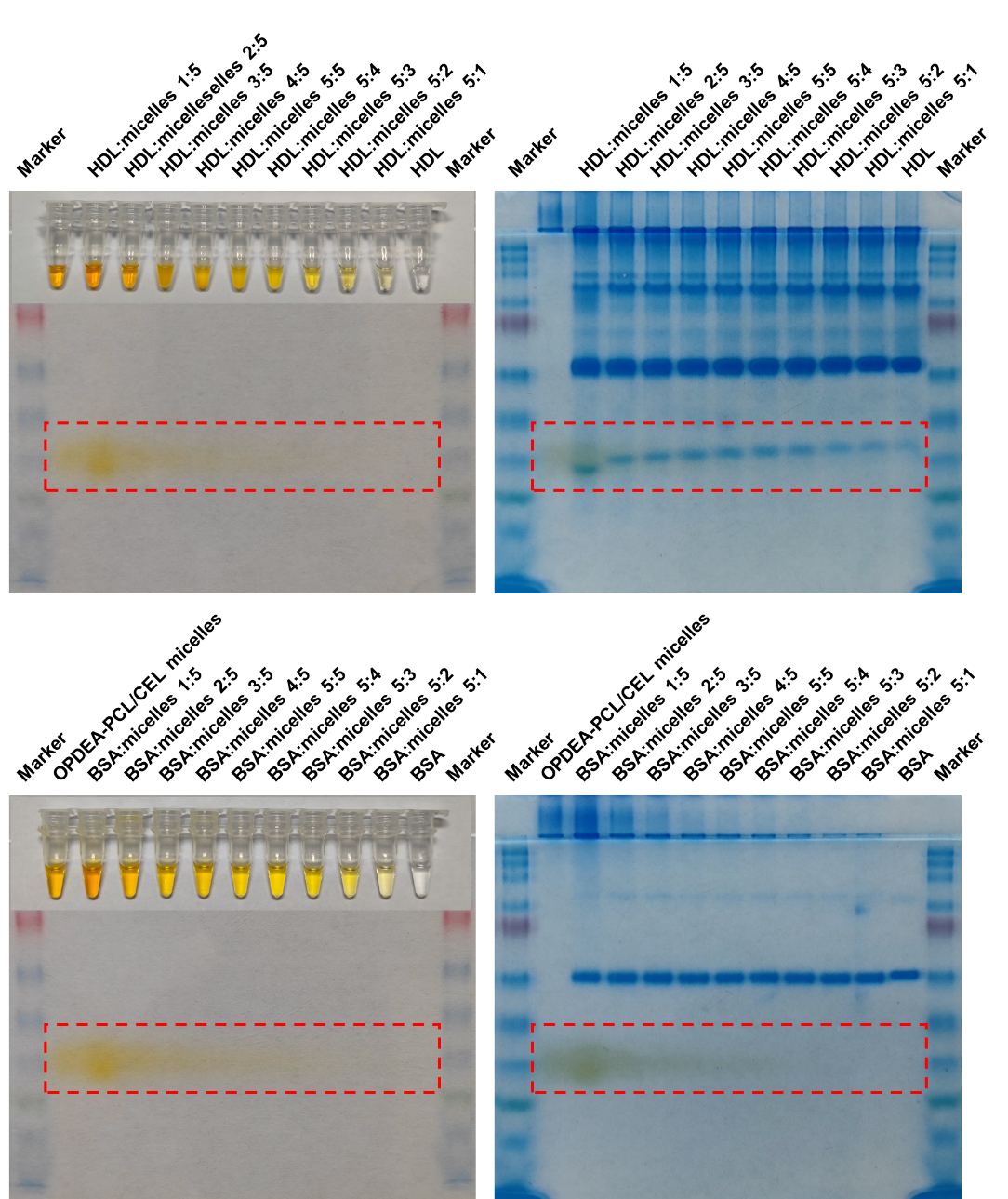


**Figure S25**. Native PAGE electrophoresis images and the corresponding photos of HDL and BSA after adding OPDEA-PCL/CEL micelles at different mass ratios. The left image showed the state before Coomassie Brilliant Blue staining, while the right showed the state after staining.


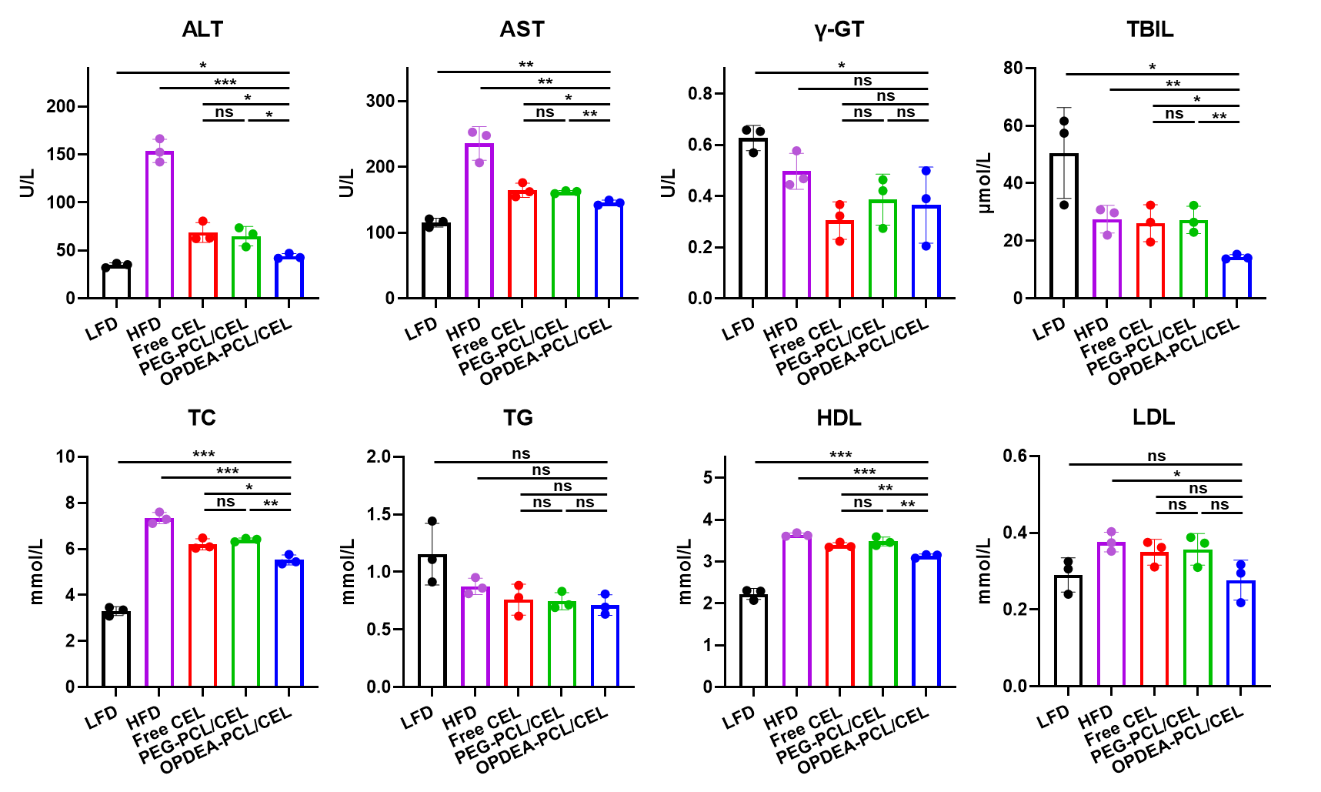


**Figure S26**. Serum biochemistry analysis of ALT, AST, γ-GT, TBIL, TC, TG, HDL and LDL levels in MASLD mice after oral treatments (n = 3).


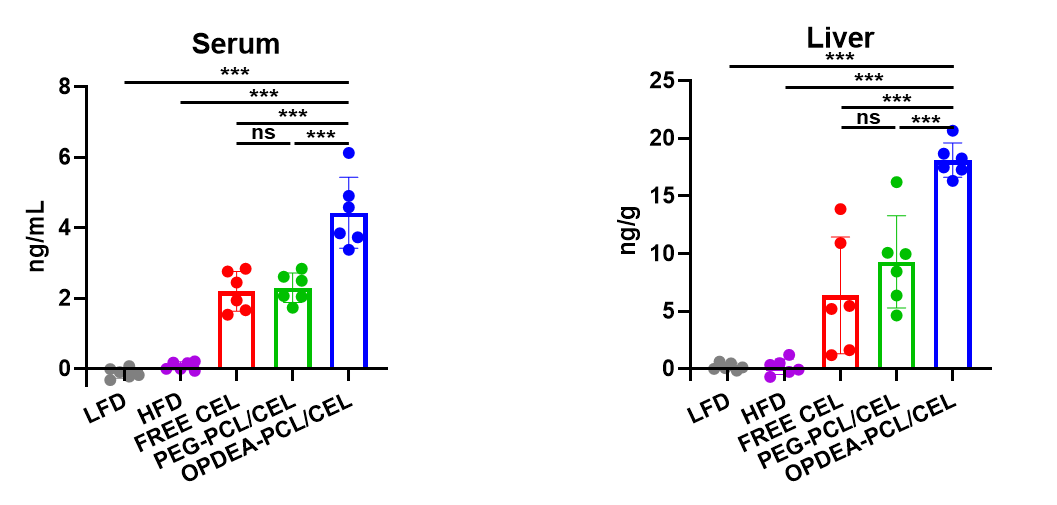


**Figure S27**. LC-MS results and quantitation of CEL content in serum and liver at the end of treatments (n = 6).


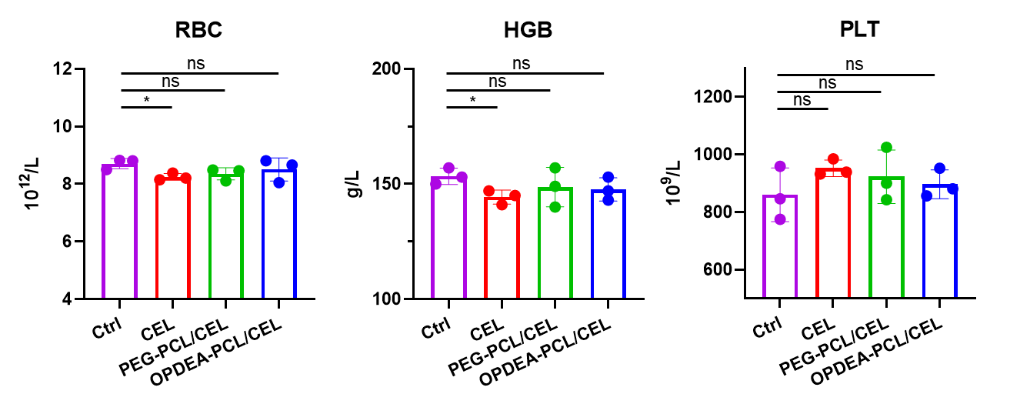


**Figure S28**. Complete blood count analyses of red blood cell (RBC), hemoglobin (HGB) and platelet (PLT) in normal mice after oral administrations (n = 3).


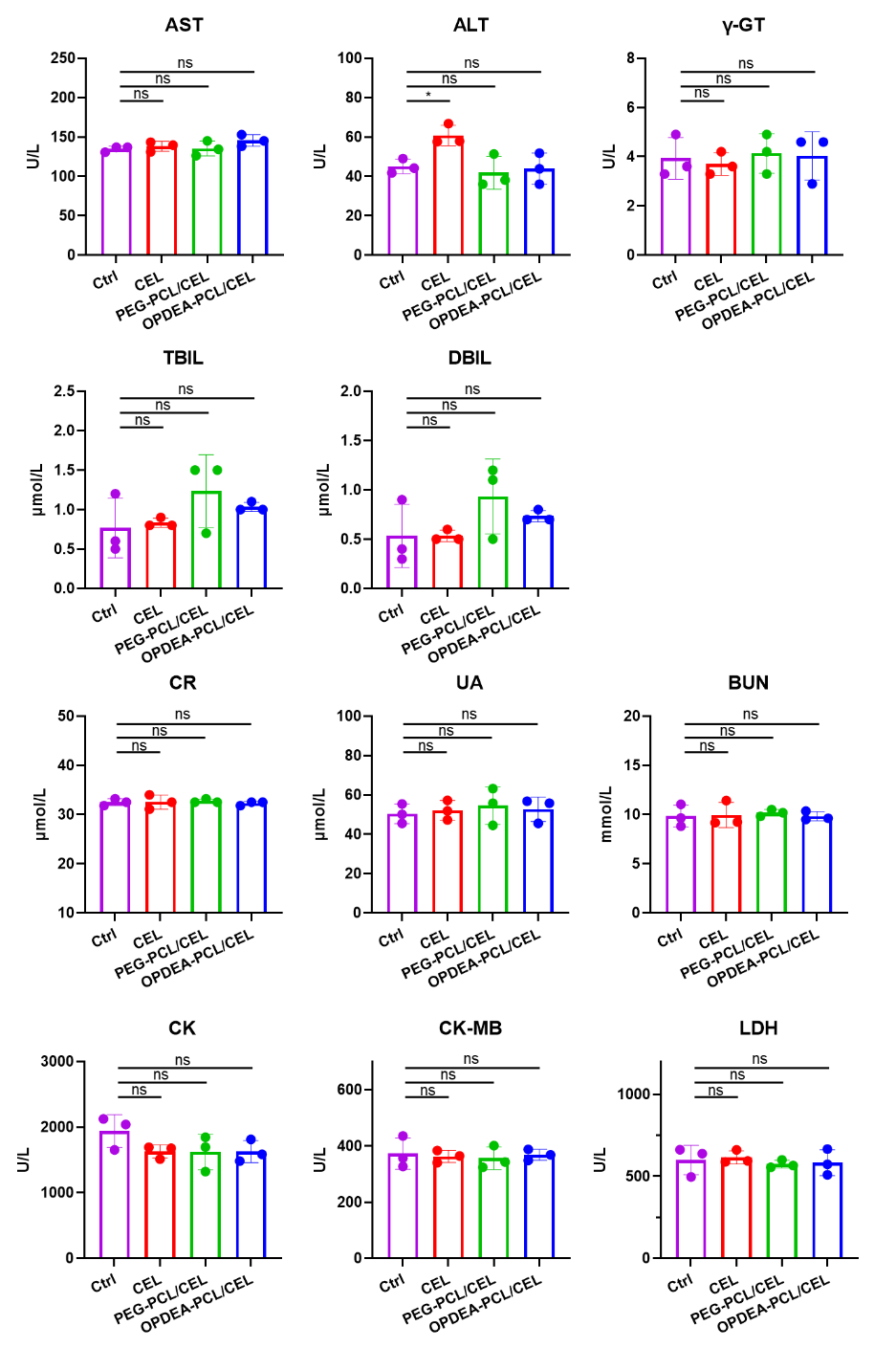


**Figure S29**. Serum biochemistry analysis of liver functions (AST, ALT, γ-GT, TBIL, DBIL), renal functions (CR, UA, BUN) and cardiac injury markers (CK, CK-MB, LDH) of normal mice after oral administrations (n = 3).


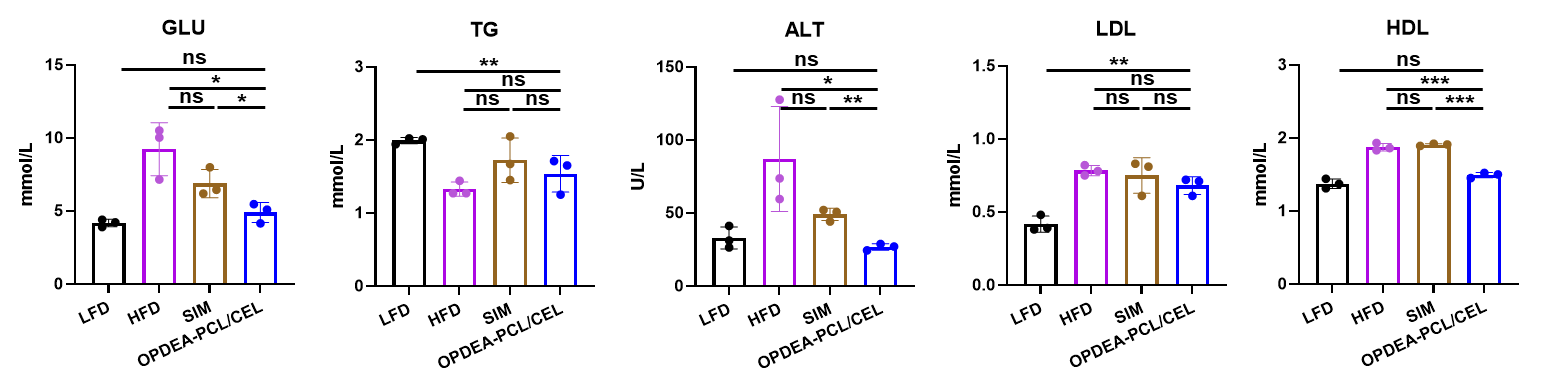


**Figure S30**. Serum biochemistry analysis of GLU, TG, ALT, LDL, HDL of MASLD mice after oral administrations (n = 3).

**Table S1.** Antibodies used in the study.

| **Antibodies** | **Company** | **Clone** | **Cat. NO.** | **Application** |
| --- | --- | --- | --- | --- |
| Fixable Viability Stain 780 | BD Horizon™ | - | 565388 | Flow |
| BV510 CD45 | BD Horizon™ | 30-F11 | 563891 | Flow |
| PE F4/80 | Biolegend | QA17A29 | 157303 | Flow |
| PE/Cyanine7 CD11b | Biolegend | M1/70 | 101216 | Flow |
| BV605 CD31 | BD OptiBuild™ | 390 | 740356 | Flow |
| Rat Anti-Mouse CD16/CD32 | BD Pharmingen™ | 2.4G2 | 553142 | Flow |
| F4/80 | Cell Signaling | D4C8V | 30325S | IF |
| HNF-4-alpha | Abcam | EPR16885-99 | ab201460 | IF |
| Goat anti-Rabbit IgG AF488 | Abcam | - | ab150077 | IF |
| phospho-AMPK | Cell Signaling | 40H9 | 2535S | WB |
| AMPK | Cell Signaling | D5A2 | 5831S | WB |
| Sirt1 | Cell Signaling | 1F3 | 8469S | WB |
| FASN | Cell Signaling | C20G5 | 3180S | WB |
| NF-κB | Cell Signaling | D14E12 | 8242S | WB |
| SREBF1 | ABclonal | - | A15586 | WB |
| PPAR-γ | ABclonal | - | A11183 | WB |
| GAPDH | Cell Signaling | 14C10 | 2118S | WB |
| β-Tubulin | Cell Signaling | 9F3 | 2128S | WB |
| HRP Goat Anti-Mouse IgG | Fdbio science | - | FDR007 | WB |
| HRP Goat Anti-Rabbit IgG | Fdbio science | - | FDM007 | WB |

Flow: Flow cytometry IF: Immune fluorescence staining WB: Western blot

**References**

[1] a)T. Fang, H. Wang, X. Pan, P. J. Little, S. Xu, J. Weng, *Int J Biol Sci* **2022**, 18, 5681; b)P. Xie, Y. Peng, L. Qiu, *Carbohydr Polym* **2022**, 288, 119388.

[2] W. Fan, Q. Wei, J. Xiang, Y. Tang, Q. Zhou, Y. Geng, Y. Liu, R. Sun, L. Xu, G. Wang, Y. Piao, S. Shao, Z. Zhou, J. Tang, T. Xie, Z. Li, Y. Shen, *Adv Mater* **2022**, 34, e2109189.
